# Tumor-TME Bipartite Landscape of PD-1/PD-L1 in Endometrial Cancers

**DOI:** 10.3390/ijms241311079

**Published:** 2023-07-04

**Authors:** Raed Sulaiman, Pradip De, Jennifer C. Aske, Xiaoqian Lin, Adam Dale, Nischal Koirala, Kris Gaster, Luis Rojas Espaillat, David Starks, Nandini Dey

**Affiliations:** 1 Department of Pathology, Avera Cancer Institute, Sioux Falls, SD 57108, USA; 2 Translational Oncology Laboratory, Avera Cancer Institute, Sioux Falls, SD 57108, USAxiaoqian.lin@avera.org (X.L.); adam.dale@avera.org (A.D.); nischal.koirala@osumc.edu (N.K.); 3 Department of Internal Medicine, University of South Dakota SSOM, Sioux Falls, SD 57108, USA; 4 Viecure, Greenwood Village, CO 80111, USA; 5 Assistant VP Outpatient Cancer Clinics, Avera Cancer Institute, Sioux Falls, SD 57108, USA; 6 Department of Gynecologic Oncology, Avera Cancer Institute, Sioux Falls, SD 57108, USA

**Keywords:** PD-1-PD-L1/L2 landscape in tumor and TME, tripartite dialogue between tumor-TME-blood, plasma PD-1, plasma PD-L1/L2, tumor-adjacent normal tissue, cancer-associated fibroblasts, CAF-PD-L1

## Abstract

The bipartite landscape of tumor cells and stromal cells determines a tumor’s response to treatment during disease management. In endometrial cancers (ECs), the mechanistic contribution of PD-L1/L2 and PD-1 signaling of the host’s tumor microenvironment (TME) (CAF and immune cells) in the context of the tumor cells is elusive. To understand the tumor–stroma-immune crosstalk, we studied the compartmental pattern of PD-L1/L2 and PD-1 expression in EC tissues and their matched CAFs. Over 116 surgically resected tumors (T) and the tumor-adjacent normal tissues (N) were obtained from consented unselected consecutive patients. IHC was performed in T, N-epi-thelium, and the stromal mesenchymal environment (SME; mesenchyme) in the T and N tissues. The staining intensity and distribution patterns of PD-L1/L2 and PD-1 in the FFPE sections of T and N were evaluated by a pathologist using a standard scoring system of TPS and CPS. We tested the PD-L1/L2 and PD-1 immune landscape of tumor-TME pair and normal epithelial-stromal mesenchyme pairs from patients with different grades of disease vis-à-vis their CAF PD-L1 levels. We used qRT-PCR to determine the expressions of mRNAs, while the flow cytometry and ICC determined the level of expression of proteins. We observed higher levels of PD-L1 mRNA and protein expression in primary CAFs from the resected tumor tissue compared to the tumor-adjacent normal tissues. We also determined the expression of patients’ soluble PD-L1/L2 as peripheral readouts of PD-L1/L2 and PD-1. As we evaluated the results in the context of their pathological parameters, such as grades, stages, lymphovascular invasion, percentage of myometrial invasion, and dMMR in patients, the dominance of PD-L1 expression in TME was positively correlated to the higher pathological grades of tumors, and its relationship with the dMMR. Since the neutralization of CD8-positive cytotoxic T-cells is PD-L1-dependent, our data indicate that irrespective of the PD-L1 positivity of tumor cells, the PD-L1-positive CAFs can play a critical role in bringing out an additional load of PD-L1 for an effective engagement of PD-1 within a tumor mass.

## 1. Introduction

Tumor cells exist as a part of thetumor–host cell ecosystem, implying the significance of the tumor cell tumor–stroma bipartite landscape in determining the response to a particular treatment for managing the disease. The coexistence of tumor cells within the TME (tumor microenvironment) and their coevolution with cells of TME, including endothelial cells, immune cells, and cancer-associated fibroblasts (CAFs), completes the entirety of the tumor–stroma bipartite landscape.

Immune checkpoint inhibition (ICI) has revolutionized the clinical management of solid tumors in the past decade [1]. PD-1 (Programmed cell death protein 1)/PD-L1 (Programmed Death-1 ligand) interaction is the basis for the therapeutic blockade by ICI with mAbs. PD-1/PD-L1 checkpoint inhibitors block the attenuation of T-cell immune function involving PD-1 on CD8+ T-cells or one of its principal counter ligands, PD-L1, on tumor cells and host immune cells. Hence ICI is an effective treatment option, as trials have been established in patients with a wide range of solid tumors, including endometrial cancers (ECs). 

Although the initial reports indicated that PD-L1 expressed in both tumor cells and non-tumor host cells contributes to PD-L1/PD-1 checkpoint blockade therapies [2,3], the host expression of PD-L1 has been shown to contribute to the efficacy of PD-L1 pathway blockade-mediated tumor regression. Recent insight by Lin et al. into the cellular mediators involved in the PD-L1 and PD-1 signaling pathways revealed a deterministic role of host PD-L1 within the host TME. Thus, it is indispensable for the therapeutic efficacy of anti–PD-L1 therapy [4]. Likewise, PD-L1 in tumor cells was mainly found to be dispensable for the response to checkpoint blockade, in contrast to PD-L1 in host myeloid cells, which were essential for the checkpoint blockade response [5].

CAFs constitute the most abundant and influential cells of the TME in most solid tumors [6,7]. CAFs are a deterministic element of the tumor ecosystem because of their profoundly critical interactions with tumor cells and host immune cells of the TME in most solid tumors [6]. In deterring the immune surveillance of tumor cells by PD-1-PD-L1-mediated signaling checkpoints, CAFs have been reported to interact with the TME’s immune landscape [8] and thus form the pro-tumorigenic support system of a progressive tumor. Clinical trials have been conducted to test the diagnostic value of CAFs and the therapeutic role of PD-1-PD-L1 immune-checkpoint inhibitors to regulate CAF-mediated immune evasion in several solid tumors [9]. Although the role of CAF is well studied and reported in various solid tumors, wherein the information provides the essential groundwork for the PD-1-PD-L1-based immune therapy [10], the PD-1-PD-L1 expression landscape remains uncharted territory in EC. To this end, we have recently reviewed the functional characteristics of CAFs in light of their dialogue with tumor cells and other components of the SME (stromal microenvironment) to find the importance of CAFs in EC [8].

Although ICI has been shown to improve disease outcomes in patients with EC [11], it has not been as effective as expected (the ORR (objective response rate) varied from 42% to 57%) even in highly immunogenic (high TMB, MMR deficiency, PD-L1 positivity) tumors [12,13]. The limitation in response to an ICI in a subset of patients suggests the unmet need to improve the effectiveness of this treatment via a deeper understanding of the mechanistic biomarkers present in tumor cells and the cells of the TME. 

The mechanistic contribution of PD-L1/L2 and PD-1 signaling of the host’s tumor microenvironment (TME) (CAF and immune cells) in the context of the tumor cells is elusive in endometrial cancers. To understand the tumor–stroma-immune crosstalk, we studied the compartmental pattern of PD-L1, PD-L2, and PD-1 expression in endometrial cancer tissues from over 116 consenting patients with grades 1, 2, and 3 diseases vis-à-vis PD-L1 expression in their matched cultured CAFs. We observed higher levels of PD-L1 mRNA and protein expression in primary CAFs from the resected tumor tissue compared to the tumor-adjacent normal tissues. We also determined the expression of patients’ soluble PD-L1/PD-L2 as peripheral readouts of PD-L1/PD-L2 and PD-1.

Considering the undeniable role of CAFs in tumor progression and treatment resistance, we hypothesized that the endometrial CAFs contribute to the PD-L1–PD-1 axis via the expression of PD-L1. To test our hypothesis, we mapped the PD-L1 landscape of a tumor, the TME, as compared to the tumor-adjacent normal epithelium and stromal microenvironment (the SME, including lymphocytes, macrophages, fibroblasts, and blood vessels) in endometrial cancers. To understand the PD-1–PD-L1-based tumor–stroma-immune (TSI) crosstalk within the host’s TME in EC in the context of tumor cells and tumor-derived primary CAFs, we studied the compartmental pattern of PD-L1 PD-L2 (Programmed Death-2 ligand), and PD-1 expression in EC tissues and matched tumor-adjacent normal tissues. Here we present a landscape of PD-L1/L2 and PD-1 expression in endometrial tumor tissues resolved into tumor cells and cells of the TME, including lymphocytes, macrophages, tumor-associated blood vessels, and CAFs in the light of pathological parameters, including MMR status. The PD-L1-dominant CAFs of the TME in our data strengthen the critical role of CAFs in determining the tumor immune environment in EC.

Our study is the first to present a landscape of expression of PD-L1/L2 and PD-1 in endometrial tumor tissues resolved into tumor cells and cells of the TME, including lymphocytes, macrophages, tumor-associated blood vessels, and CAFs in the light of pathological parameters, including MMR status. Since the neutralization of CD8-positive cytotoxic T-cells is PD-L1 dependent, our data indicate that irrespective of PD-L1 positivity of tumor cells, the PD-L1 positive CAFs can play a critical role in bringing out an additional load of PD-L1 for an effective engagement of PD-1 within a tumor mass. Our comprehensive data on the landscape of PD-L1/L2 and PD-1 highlight that, in addition to the tumor compartment, the CAFs of the TME bear significant immunological signatures pertaining to the PD-L1/PD-1 signaling axis, which is worth a deeper interrogation in the light of clinical relevance in patients with endometrial cancers.

## 2. Results

### 2.1. Plan of the Study

**Figure 1:**
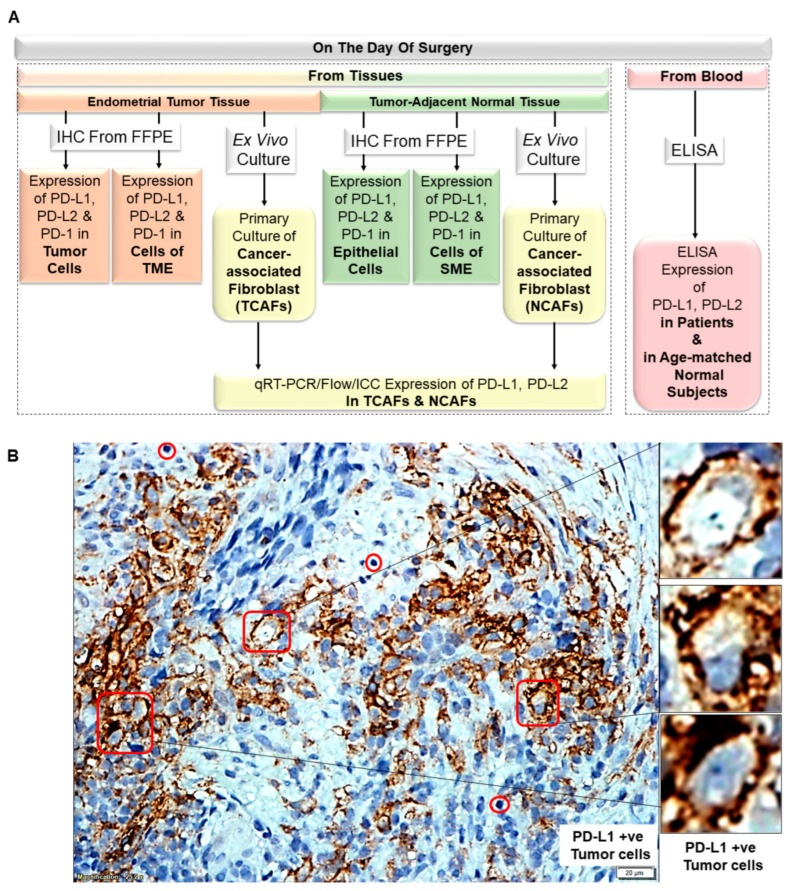

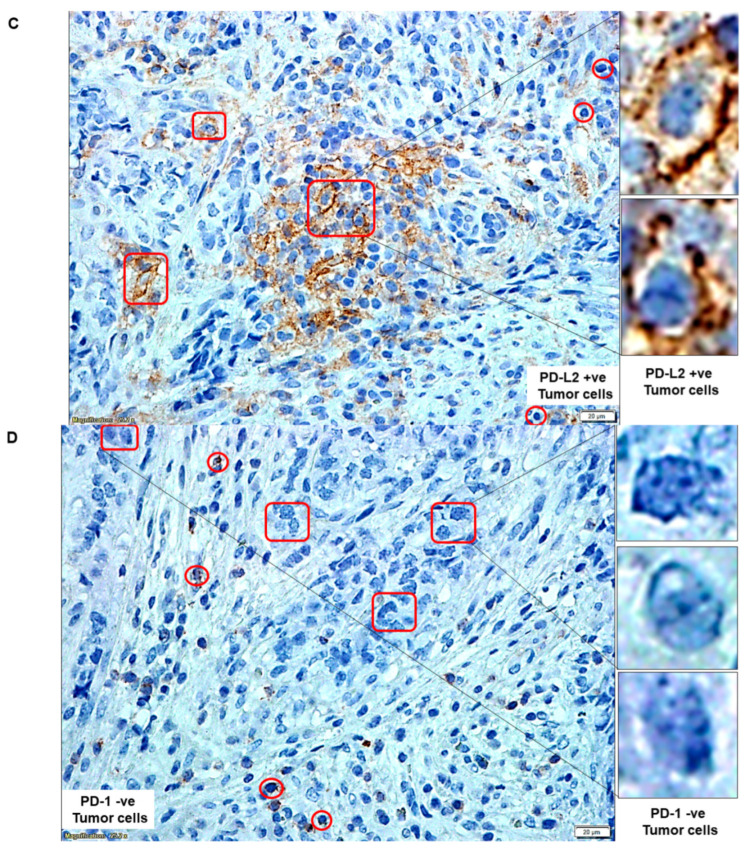

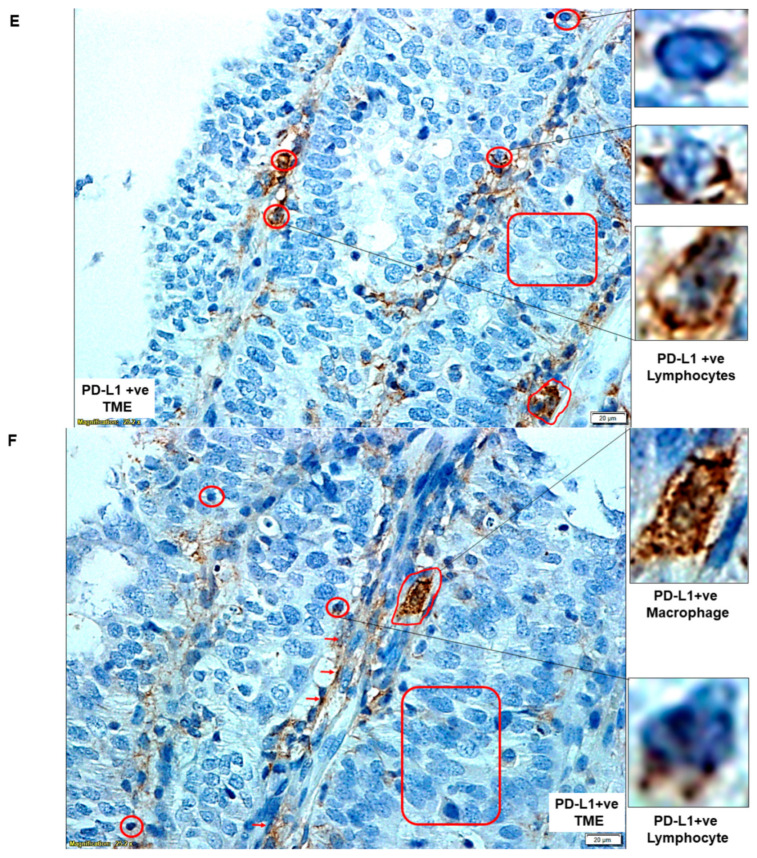

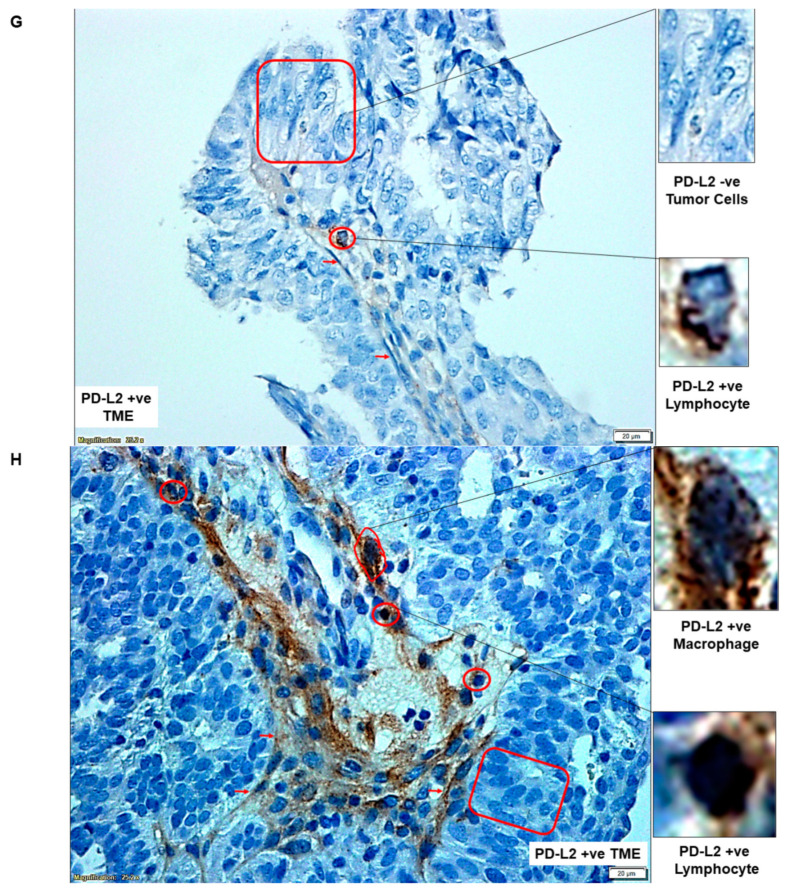

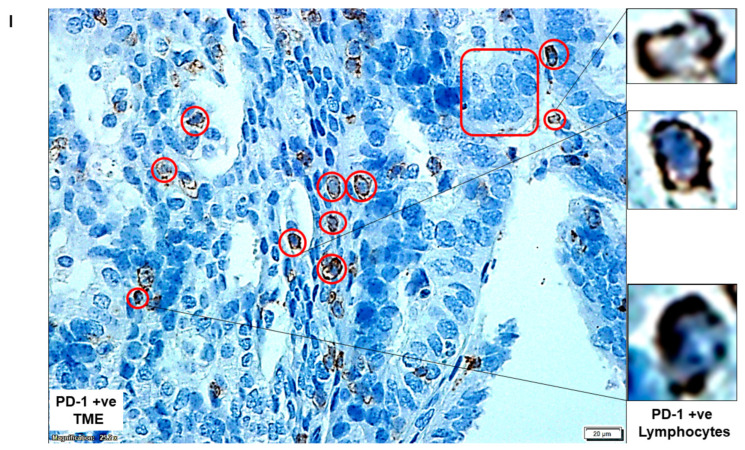
PD-L1/PD-L2 and PD-1 status of tumor cells and cells of the tumor microenvironment, TME (lymphocytes, L; macrophages, M; and blood vessels, BV) from tumor tissues: (**A**): Plan of the study for obtaining PD-L1/PD-L2 and PD-1 status of tumor cells’ tumor-adjacent normal tissue, cells of the tumor microenvironment, TME (lymphocytes, L; macrophages, M; and blood vessels, BV), and cells of the stromal microenvironment, SME, of patients with endometrial cancers. (**B**–**D**): Expression of PD-L1/PD-L2 and PD-1 in tumor cells from IHC of FFPE sections. (**E**–**I**): Expression of PD-L1/PD-L2 and PD-1 in cells of the TME (lymphocytes, macrophages, and blood vessels) from FFPE of tumor tissues. The red square is a tumor cell; the red circle is a lymphocyte (L); the red arrow is a blood vessel (BV); the red freeform is a macrophage (M). Insets show images of the selected cells at higher magnifications. Bars represent 20 µM.

The study presented here is an associated part of a patent application (United States Patent and Trademark Office; Application number 16/875,910). The study plan (Figure 1A) involved obtaining surgically resected tumors and tumor-adjacent normal tissues from patients with EC on the day of surgery. We also obtained blood samples on the day of surgery. Both the resected tumor and tumor-adjacent normal tissues were used for two purposes: (1) to evaluate the expression of PD-L1, PD-L2, and PD-1 in tumor cells, in cells of the tumor microenvironment (TME), in epithelial cells, and in cells of the stromal microenvironment (SME); and (2) to derive ex vivo primary culture of cancer-associated fibroblasts, TCAFs (CAFs derived from tumor tissues), and NCAFs (CAFs derived from tumor-adjacent normal tissues) from the tumor tissue and tumor-adjacent normal tissues, respectively. The microscopic evaluation of PD-L1, PD-L2, and PD-1 expression was carried out as previously described [14]. We performed qRT-PCR/Flow/ICC (immuno-cytochemistry) to test the expression of PD-L1 and PD-L2 in cultured TCAFs and NCAFs. Blood was used to test the presence of sPD-L1 (soluble PD-L1) and sPD-L2 (soluble PD-L2) in patients and age-matched healthy subjects by ELISA. We first determined the expression pattern of PD-L1/2 and PD-1 in tumor cells and cells of the TME from tumor tissue samples and compared it with the tumor-adjacent normal tissues. We hypothesized that the CAFs are one of the critical sources of PD-L1 in EC. To test our hypothesis, we generated patient-derived CAFs and tested the expression of PD-L1/2 by qRT-PCR, flow cytometry, and ICC. To know whether the expression of PD-L1 in the tumor is reflected in the peripheral blood, we also examined the sPD-L1/2 in the blood of patients with EC and compared it with the blood of healthy normal subjects. 

### 2.2. PD-L1/PD-L2 and PD-1 Protein Expression Dominated in Tumor Tissues as Compared to the Paired Samples of Tumor-Adjacent Normal Endometrial Tissues

The staining intensity and distribution pattern were evaluated by a pathologist using a standard scoring system of TPS (Tumor Proportion Score; 1% cut-off value) and CPS (Combined Proportion Score; 1 cut-off value). The overall expression pattern of PD-L1/PD-L2 and PD-1 proteins in the paired samples demonstrated a clear differential presence in the tumor tissues as compared to the tumor-adjacent normal tissues. The presence of PD-L1/PD-L2 and PD-1 proteins was significantly higher in the tumor tissues and both tumor cells (EpCAM+/CK8, 18+), as well as in the cells of the TME, including lymphocytes (CD3+/CD4+/CD8+), and macrophages (CD68+/CD163+) as compared to the epithelial cells and cells of SME within the tumor-adjacent normal tissues where only focal positivity was observed rarely. 

Figure 1B–I presents representative photomicrographs of PD-L1/PD-L2 and PD-1 expression of tumor cells and cells of the TME of tumor tissues from patients with grade 3 stage 1 ((carcinosarcoma predominantly endometrioid) node-positive poorly differentiated EC with 48% myometrial invasion, LVI, and pT1a (sn)pN0i+). Both tumor and the TME are positive for both PD-L1 and PD-L2 and, as expected, negative for PD-1 (Figure 1D). Figure 1B,C show the distinct and continuous membranous staining for PD-L1 and PD-L2 of the tumor cells. In contrast, lymphocytes and macrophages of the TME of tumor tissues from patients with grade 1 stage 1 ((invasive endometrioid adenocarcinoma) node-negative EC with 32% myometrial invasion, LVI absent, and pT1a) were discretely positive for both PD-L1 and PD-L2 (Figure 1E–H). Figure 1I shows PD-1 positivity in lymphocytes in the same patient. 

**Figure 2:**
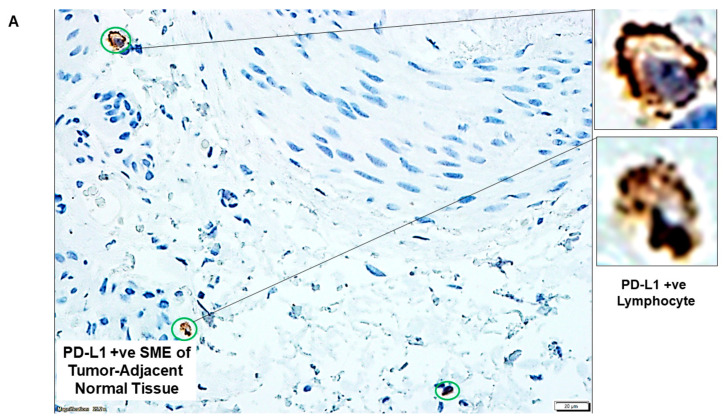

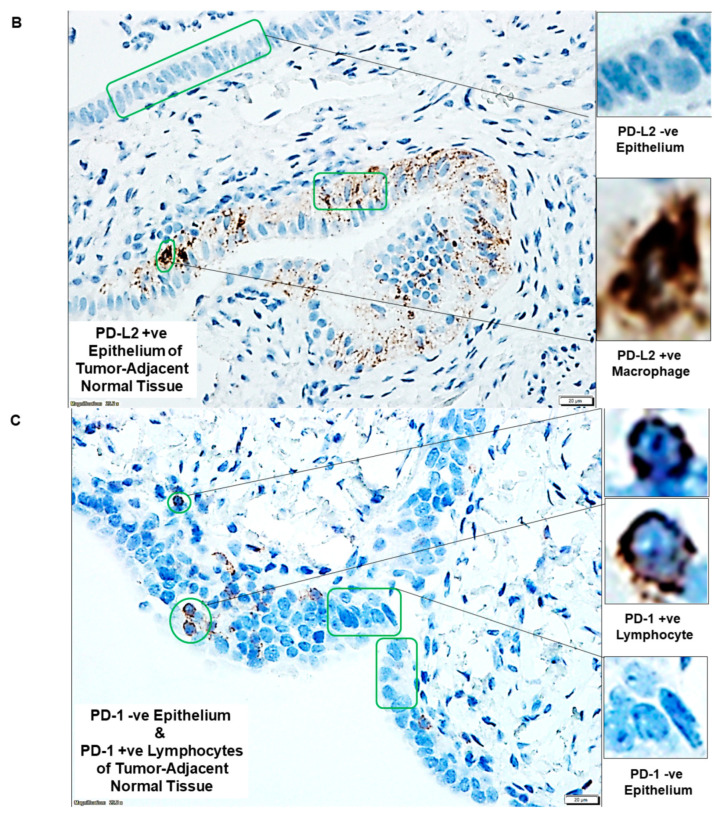

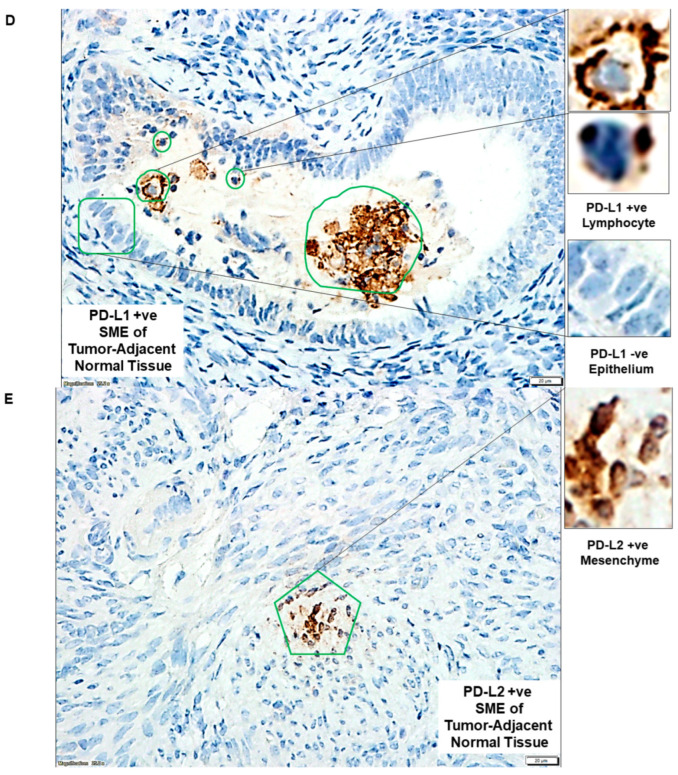

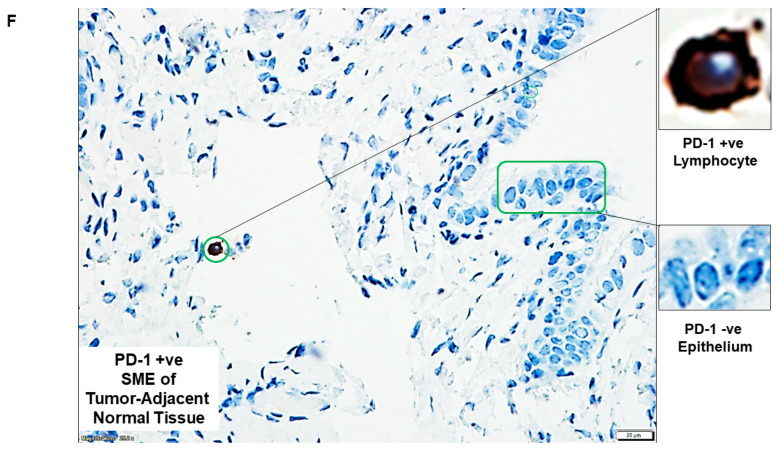
PD-L1/PD-L2 and PD-1 status of epithelial cells and cells of the stromal microenvironment, SME (lymphocytes, L; macrophages, M;) from tumor-adjacent normal tissues: (**A**–**F**): Expression of PD-L1/PD-L2 and PD-1 by IHC of FFPE sections from epithelial cells of tumor-adjacent normal tissues. The expression of PD-L1/PD-L2 and PD-1 in mesenchymal cells of the stromal microenvironment, SME (lymphocytes, macrophages, and blood vessels) from FFPE sections by IHC is presented. The green square/rectangle is an epithelial cell; the green circle is a lymphocyte (L); the green freeform is a macrophage (M); the green pentagon represents mesenchyme. Insets show images of the selected cells at higher magnifications. Bars represent 20 µM.

Figure 2 presents representative photomicrographs of PD-L1/PD-L2 and PD-1 expression of epithelial cells and cells of SME of tumor-adjacent normal tissues from three patients. Figure 2A,B show the expression of PD-L1 and PD-L2, respectively, in the epithelium of the tumor-adjacent normal tissues obtained from a patient with grade 1 stage 1 endometrioid adenocarcinoma (a node-negative disease with 0% myometrial invasion, no LVI, and pT1a score). Figure 2C,F show the absence of PD-1 in the epithelium and also in the SME in tumor-adjacent normal tissues from a patient with grade 2 stage 1 endometrioid adenocarcinoma (a node-negative disease with 11% myometrial invasion, no LVI, and pT1a score). Figure 2D,E show the expression of PD-L1 and PD-L2, respectively, in the SME of tumor-adjacent normal tissues from this patient. 

Overall, the expression of three immune checkpoint proteins was limited in tumor-adjacent normal tissues both in the epithelial and mesenchymal compartments. Only rare and focal positivity was observed in PD-L1 in the mesenchymal regions and intratumoral/intraepithelial immune cells, including peritumoral lymphocytes and macrophages, and very rarely in the vicinity of glands and blood vessels. The tumor compartments, in contrast, were positive for PD-L1 in 70% of the tested samples, which was independent of differentiation status, presence of TILs, or LVI (lymphovascular invasion). In comparison, the TME components were positive for PD-L1 in 90% of tested samples. The stromal positivity of PD-L1 was identified predominantly in (1) the immune component, including macrophages, followed by lymphocytes, and (2) the mesenchymal component, EpCAM-/SMA+/FAP+/S100A4+ CAFs. Both PD-L1 and PD-L2 were identified by flow, ICC, and qRT-PCR in NCAF and TCAF. The overall expression of PD-L2 was found to be qualitatively and quantitatively less than that of PD-L1, primarily contributed by SME/stromal macrophages and lymphocytes. PD-1 expression was restricted to the SME/stromal immune components, predominantly lymphocytes. PD-L1 and PD-L2 mRNA expressions were higher in TCAF than in NCAF in successive passages, which ICC and flow cytometry confirmed. The expression of PD-L1 and PD-L2 is primarily tumor-driven and associated with invasive endometrioid and serous adenocarcinoma and, more commonly, with myometrial invasion. PD-L1 and PD-L2 proteins are distributed in both tumor and SME/stromal components, while PD-1 expression is exclusively SME/stromal.

### 2.3. PD-L1 Dominated TME Landscape in Endometrial Tumor Tissues 

As mentioned before, PD-L1 and PD-L2 expressions were identified by flow, ICC, and qRT-PCR in NCAF and TCAF. Overall, the tumor cell compartments of tumor tissue had 70% positivity for PD-L1, which was independent of differentiation status, presence of TILs, or LVI. PD-L1 positivity was observed in 90% of the TME, identified predominantly in the (1) immune component (macrophages and lymphocytes) and (2) EpCAM-/SMA+/FAP+/S100A4+ CAFs. The overall expression of PD-L2 was found to be qualitatively and quantitatively less than that of PD-L1, primarily contributed by macrophages and lymphocytes of the TME. PD-1 expression was restricted to the TME’s immune components, predominantly in lymphocytes. The expressions of PD-L1 and PD-L2 were distributed in both tumor and TME components, while PD-1 expression was predominantly found in the TME. In order to test the dominance of one protein over another or their differential distribution in different regions of the tumor and tumor-adjacent normal tissue, we compared the IHC expressions of PD-L1, PD-L2, and PD-1 in (1) epithelium vs. SME, (2) tumor vs. TME, (3) epithelium vs. tumor, and (4) TME vs. SME in tumor and tumor-adjacent normal tissues from 26 patients with EC (Table 1). The difference in expression of all three immune checkpoint proteins, PD-L1, PD-L2, and PD-1, between the epithelium and SME of tumor-adjacent normal tissues was found to be insignificant. In contrast, the difference in the expression of the PD-1 receptor between the tumor and the TME of tumor tissues was significant, while the difference in the expression of its ligand, PD-L1, and PD-L2 between the tumor and the TME of tumor tissues was insignificant. When compared between the epithelium of the tumor-adjacent normal tissue and the tumor cells of the tumor, the expression of both the ligands, PD-L1 and PD-L2, was found to be significant. In contrast, the expression of PD-1 was insignificant. We also compared the SME of the tumor-adjacent normal tissue and the TME of the tumor to show that the expression of all three immune checkpoint proteins, PD-L1, PD-L2, and PD-1, was significant. In summary, the expression pattern demonstrates a dominance of expression in the tumor and the TME over the epithelium and the SME of the tumor-adjacent normal tissue.

### 2.4. Establishment of Patient Tissue-Derived Primary Culture of CAF Ex Vivo

We established the primary culture of CAFs from both tumor tissues (TCAFs) and the tumor-adjacent normal tissues (NCAFs) from 53 patients with EC who consented to the study [15]. Our primary cultures of CAFs were set up from tissue sample(s) on the day of surgery(s) (within 1 h of surgical resection) in a normoxic condition. The passaged primary cultures of NCAFs and TCAFs were characterized and validated based on the markers for CAFs (positive) and negative markers of tumor/epithelial cells/endothelial cells. 

### 2.5. Marker-Based Validation of CAFs in Ex Vivo Culture of the Patient’s Tumor Tissue and Tumor-Adjacent Normal Tissue by Flowcytometry 

**Figure 3:**
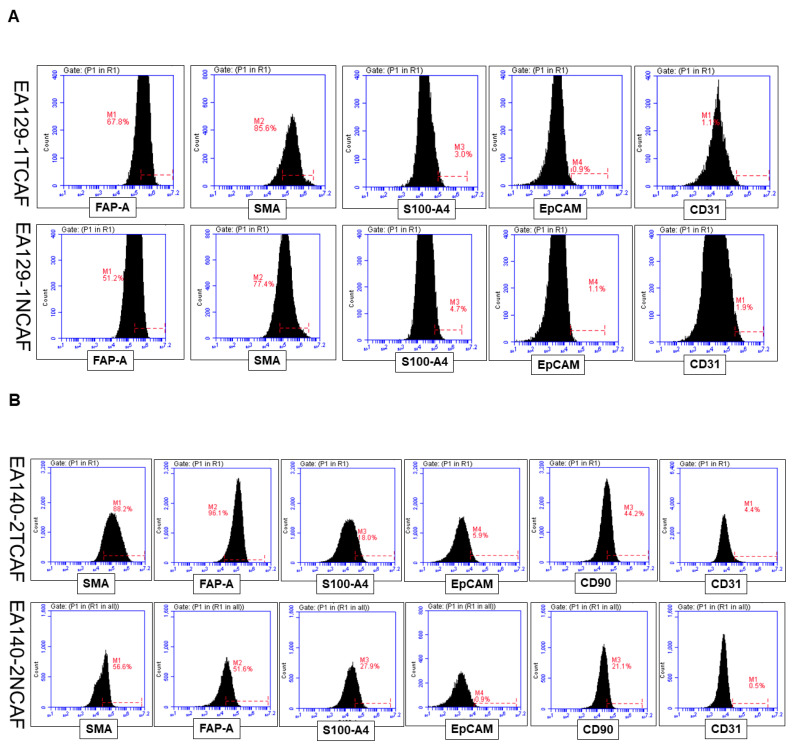

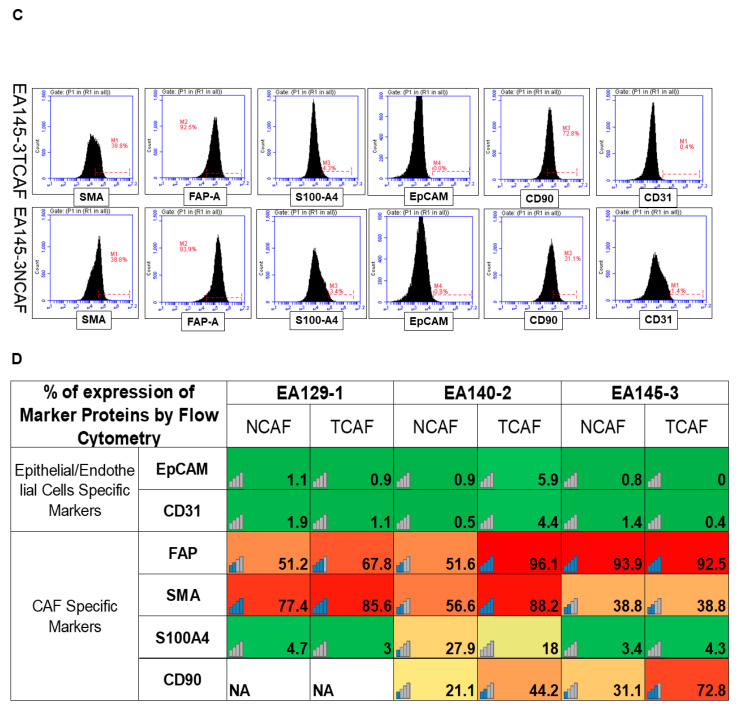

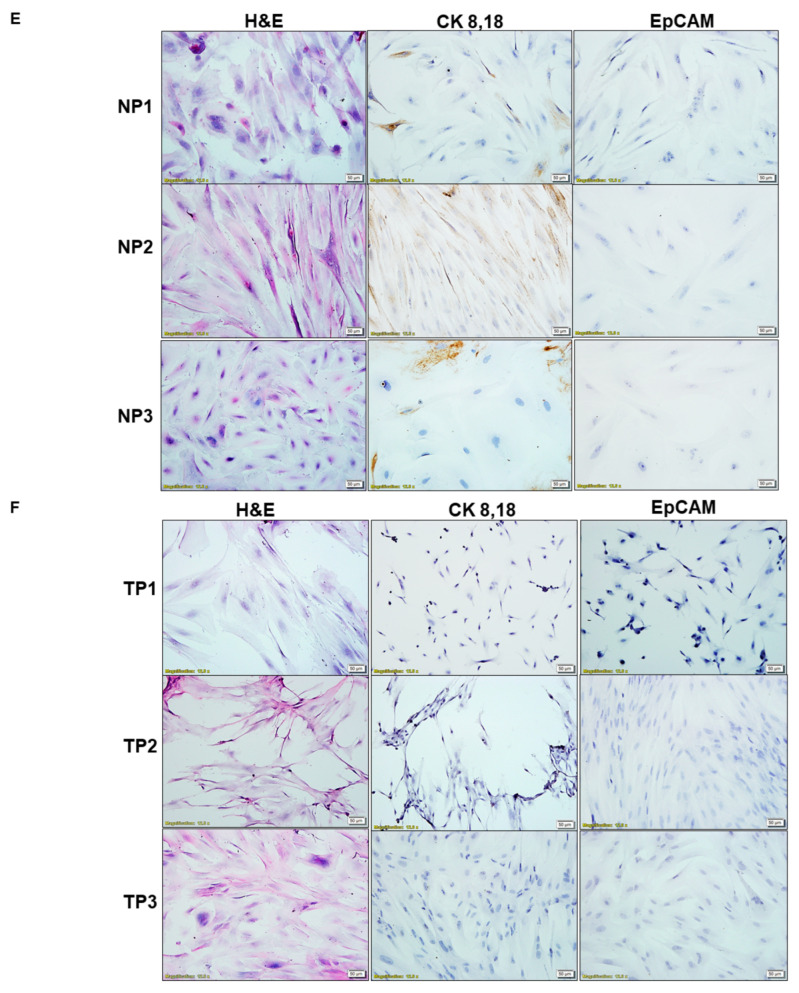

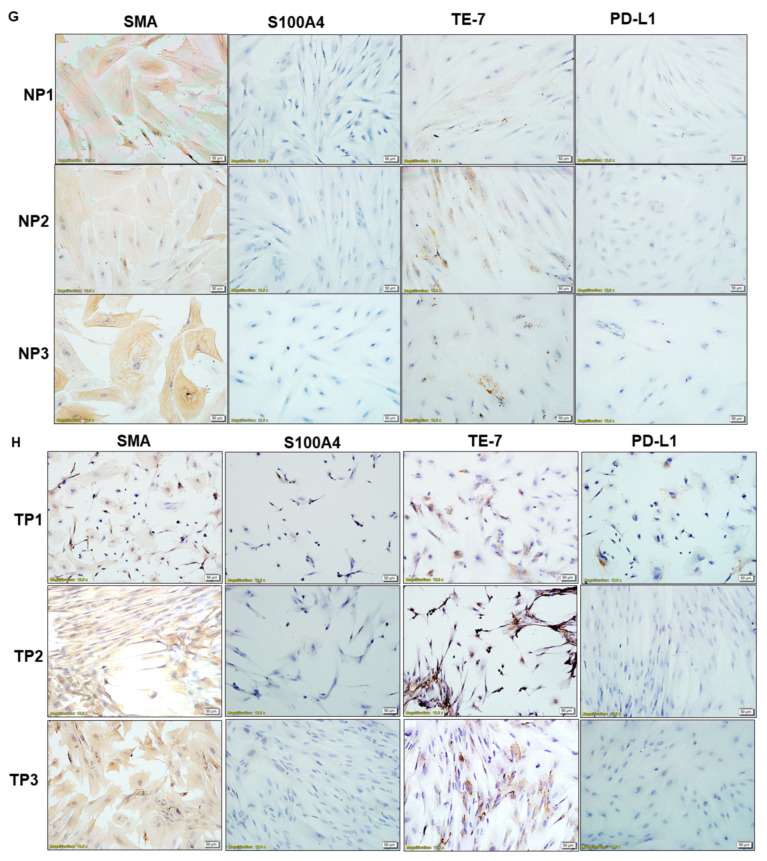

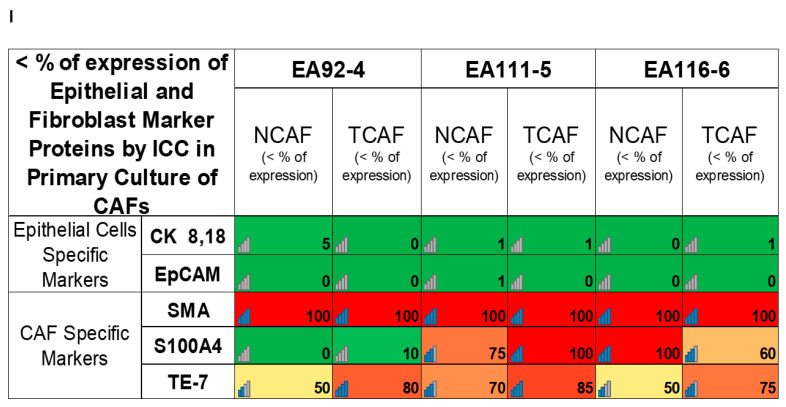
Marker-based characterization of NCAFs and TCAFs by flowcytometry and ICC: The percentage of the expression of epithelial (EpCAM), fibroblast (SMA, S100A4, FAP, and CD90), and endothelial (CD31) marker proteins by flow cytometry are presented (**A**–**D**). The data presented are from the built-in analyses program of the flow cytometer generated based on the MFI. Marker expression was set based on isotype-control(s) stained samples. The base of the scale bar presented for the flow cytometry data is 10. Expressions in cells were formatted based on their values using three-color scales (red as the highest and green as the lowest) and five-rating icon sets. CAFs represent cells from the early (mostly P1/2/3) passages. The percentage of expression of epithelial (EpCAM and CK 8, 18) and fibroblast (SMA, S100A4, and TE-7) marker proteins by ICC in primary culture of CAFs (**E**–**I**) are presented. The expression of cells was formatted based on their values using three-color scales (red as the highest and green as the lowest) and five-rating icon sets. CAFs represent cells from the early (mostly P1) passages. Both NCAFs and TCAFs are negative for both epithelial markers (**I**). Bars represent 50 µM.

We performed marker-based validation of isolated CAFs in ex vivo culture of the patient’s tumor tissue and tumor-adjacent normal tissue by flow cytometry. Figure 3 shows the pattern of distribution of % of CAF positive and negative markers in histograms from three representative tumors and tumor-adjacent normal samples in pairs (Figure 3A–C). The table (Figure 3D) summarizes and conditionally formats the % expression of CAF positive and negative markers. The percentage of expression of epithelial (EpCAM), fibroblast (SMA, S100A4, FAP, and CD90), and endothelial (CD31) marker proteins by flow cytometry in the primary culture of CAFs was determined. The expression of cells was formatted based on their values using three-color scales (red as the highest and green as the lowest) and five-rating icon sets. CAFs represent cells from the early (mostly P1/2/3) passages. Both NCAFs and TCAFs were negative for both epithelial and endothelial markers. The expression of SMA was positive in all three pairs, with a tendency of higher expression in TCAFs as compared to NCAFs in two pairs out of three. FAP expressions were found to be higher (>50%) than SMA expressions in all three pairs, with a similar tendency of higher expression in TCAFs as compared to NCAFs in two pairs out of three. The percentage expression of CD90 was found to be higher in TCAFs than in the paired NCAFs. The percentage of the expression of S100A4, in contrast, was significantly lower than the expression of SMA and FAP. Two of the three paired samples had a negligible expression of the marker. We extended our marker-based validation to test the subcellular distribution of the markers in three CAF pairs by ICC in primary cultures (Figure 3E–H). The figures present the percentage of the expression of epithelial (EpCAM and CK 8,18) and fibroblast (SMA, S100A4, and TE-7) marker proteins by ICC in the primary culture of CAFs. The expression of cells was formatted based on their values using three-color scales (red as the highest and green as the lowest) and five-rating icon sets. CAFs represent cells from the early (mostly P1) passages (Figure 3I). Both NCAFs and TCAFs were negative for both epithelial markers. Similar to the percentage of expression observed by flow cytometric analyses, the CAF pairs were negative for both epithelial markers, CK 8,18 and EpCAM (as validated using epithelial cancer cell lines from endometrial and lung cancers, RL-95-2 and NCI-H441). Similarly, 100% SMA expression was predominant across all pairs. The expression of TE-7 was equal to or higher than 50% across all pairs, with a clear tendency of higher expression in TCAFs as compared to NCAFs.

### 2.6. Stromal PD-L1 and PD-L2 Expressions in Tumor (TCAF from TME) and Tumor-Adjacent Normal (NCAF from SME) 

#### 2.6.1. PD-L1/PD-L2 mRNA Expression Status in Cultured CAF from Paired Samples of Tumor and Tumor-Adjacent Normal Tissues by qRT-PCR

**Figure 4:**
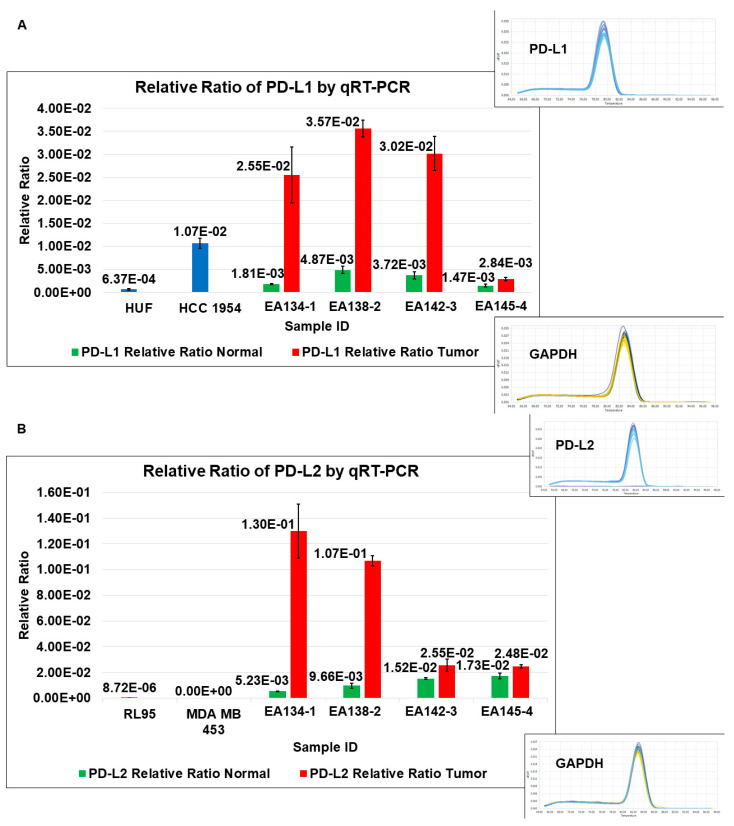

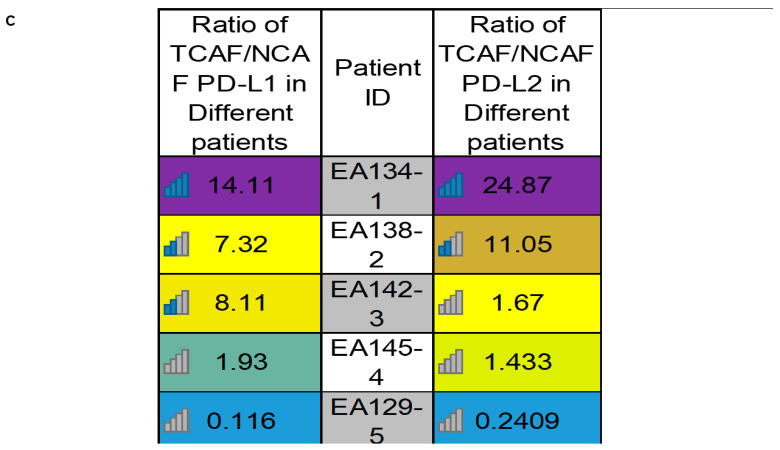
Expression of PD-L1 and PD-L2 mRNA in paired CAFs in primary culture from tumor and tumor-adjacent normal samples obtained from patients with endometrial cancers: PD-L1 (**A**) and PD-L2 (**B**) mRNA expression (relative ratios to GAPDH) of NCAFs and TCAFs were carried out by qRT-PCR. HUF and tumor cell lines were used as controls. Melting curves for the respective mRNAs and GAPDH are presented as insets. A heatmap of PD-L1 and PD-L2 mRNA expression is presented as ratios of TCAFs/NCAFs pairs (**C**) of different patients with endometrial cancers. The five-rating icon sets (bars) with a three-color scale (light blue as a minimum, yellow as the midpoint, and violet as the maximum) are used.

We determined the expression of PD-L1 and PD-L2 (PD-1 is not expressed in CAFs as expected;) in four NCAF-TCAF pairs derived from ex vivo primary cultures (Figure 4A,B). PD-L1/PD-L2 mRNA expression status in cultured CAFs from paired samples of T and N by qRT-PCR showed that both PD-L1 and PD-L2 are expressed in NCAFs and TCAFs but to a significantly different degree. TCAFs expressed significantly higher levels of both PD-L1 and PD-L2 as compared to NCAFs in three of four pairs. Heatmap of PD-L1 and PD-L2 mRNA expression presented as ratios of TCAFs/NCAFs pairs of five patients with EC using five-rating icon sets (bars) with a three-color scale (light blue as minimum, yellow as midpoint, violet as maximum) (Figure 4C) indicating patients with high and low ratios of TCAFs/NCAFs for PD-L1 and PD-L2. 

#### 2.6.2. PD-L1 Protein Expression Status in Cultured CAF from Paired Samples of Tumor and Tumor-Adjacent Normal Tissues by Flow Cytometry

**Figure 5:**
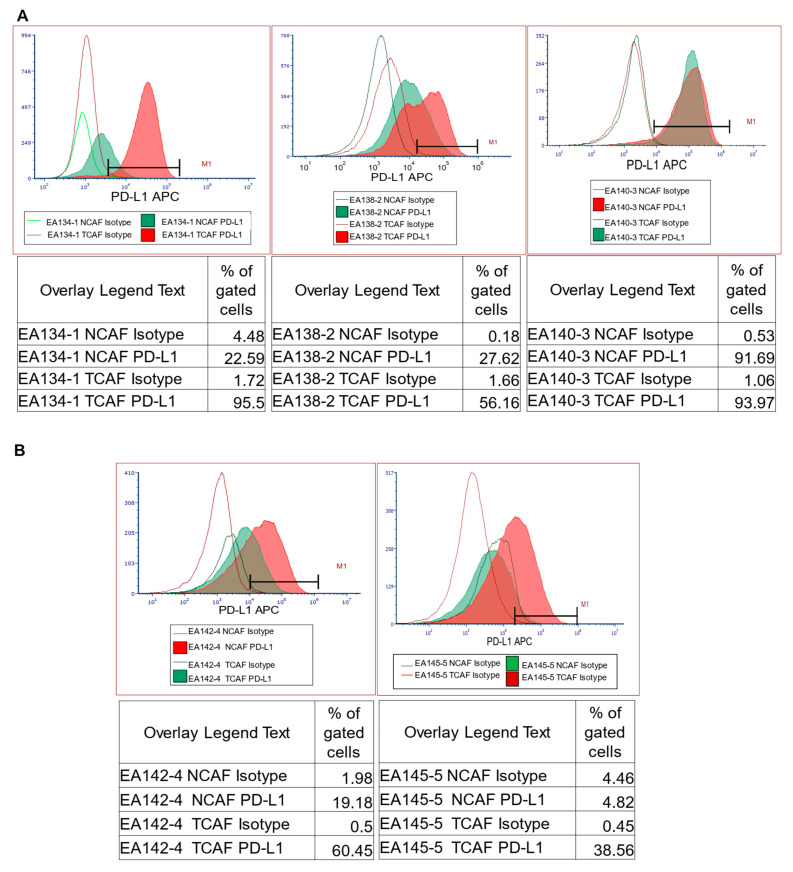

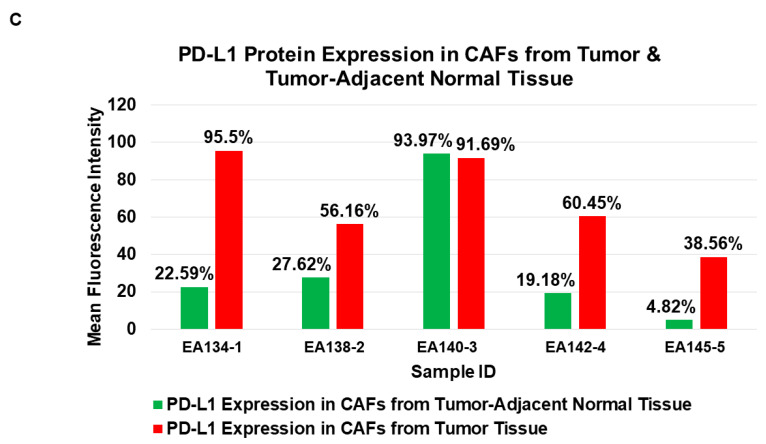
PD-L1 protein expression status in cultured CAFs from paired samples of tumor and tumor-adjacent normal tissues by flow cytometry: PD-L1 expression in five representative samples from patients with endometrial cancers. The overlay of PD-L1 expressing NCAFs (solid green area) and TCAFs (solid red area) (**A**,**B**) with isotype controls, along with the percentage of gated cells, are presented. The bar diagram of the percentage of PD-L1+ NCAFs and TCAFs are presented (**C**).

We tested the expression of PD-L1 protein in paired CAFs derived from tissues of patients with EC. Figure 5 presents the PD-L1 expression in five representative samples from patients with EC. Figure 5A,B show the overlay of PD-L1 expressing NCAFs (solid green area) and TCAFs (solid red area) with isotype controls, along with the % of gated cells. Figure 5C presents the bar diagram of % of PD-L1+ CAFs. The % expression was found to be significantly higher in TCAFs as compared to NCAFs in all but one case. We observed that the patients with no difference in the expression level of PD-L1 of NCAF and TCAF also presented significantly higher levels of PD-L1 in NCAF. 

#### 2.6.3. Cellular Distribution of PD-L1 Protein in Primary Cultured CAF from Paired Samples of Tumor and Tumor-Adjacent Normal Tissues by ICC Staining

**Figure 6:**
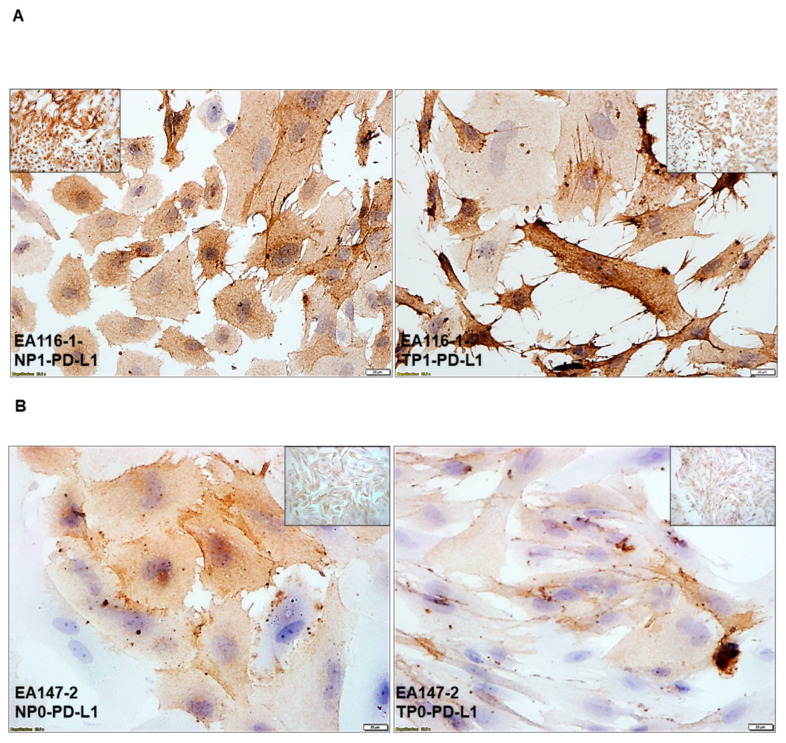

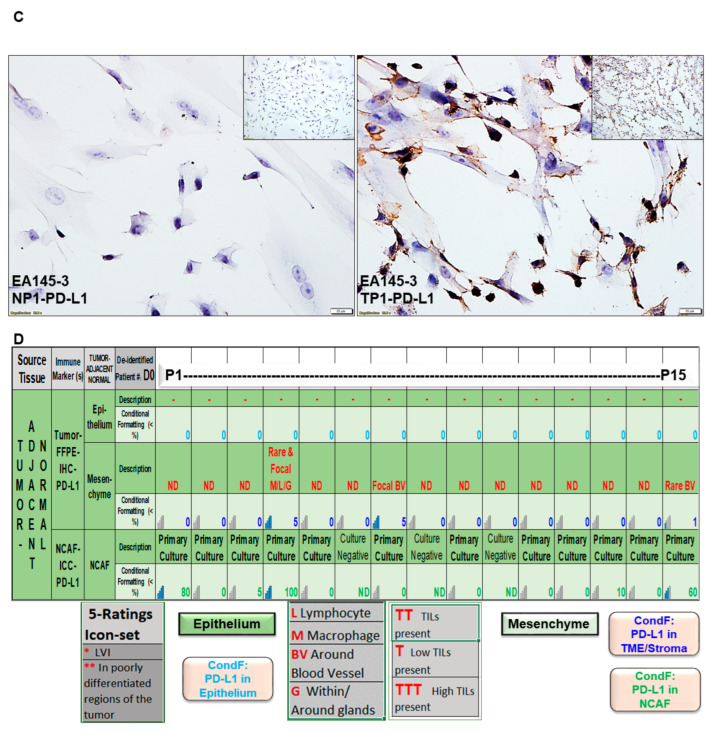

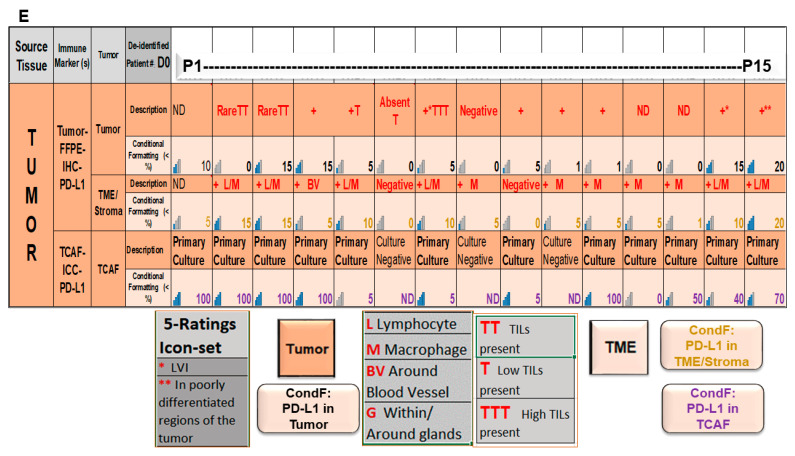
Cellular distribution of PD-L1 protein in primary cultured CAF from paired samples of tumor and tumor-adjacent normal tissues by ICC staining: Non-nuclear expression of the protein on CAFs from three representative patients’ samples with high (**A**), medium (**B**), and low (**C**) expression levels are presented. The conditional formatting by five-rating icon sets of expression of PD-L1 in tumor-adjacent normal epithelium-mesenchyme-NCAFs (**D**) and tumor-TME/Stroma-TCAFs (**E**) by IHC on FFPE sections for tissues from Day0 (day of surgery) and by ICC of tissue-derived CAFs (NCAFs and TCAFs) are presented. Bars represent 20 µM. (**D**). Conditional formatting of PD-L1 stains by five-rating icon sets of tumor-adjacent normal tissues and SME, including lymphocytes, macrophages, and around blood vessels, in lighter green rows are presented. (**E**). Conditional formatting of PD-L1 stains by five-rating icon sets of tumor cells and cells of TME (numbers in green font represent % of positive cells in TME, including lymphocytes, macrophages, and around blood vessels in lighter orange rows) are presented. Semi-quantification of TILs is represented in the figure. * represents lymphovascular invasion, and ** represents poorly differentiated regions of the tumor. Each column represents data from tumor-adjacent normal tissue (**D**) and tumor tissue (**E**) from an individual patient.

We tested the cellular distribution of PD-L1 protein by ICC staining in CAFs from paired tumor and tumor-adjacent normal tissue samples. Figure 6 shows the non-nuclear expression of the protein on CAFs from three representative patient samples with high (Figure 6A), medium (Figure 6B), and low (Figure 6C) expression levels. Conditional formatting by five-rating icon sets of expression of PD-L1 in tumor-adjacent normal epithelium-mesenchyme-NCAFs (Figure 6D) and tumor-TME/Stroma-TCAFs (Figure 6E) by IHC on FFPE sections for tissues from Day0 (day of surgery) and by ICC of tissue-derived CAFs (NCAFs and TCAFs) are presented. Conditional formatting of PD-L1 stains by five-rating icon sets of tumor-adjacent normal tissues and SME, including lymphocytes, macrophages, and around blood vessels, in lighter green rows (Figure 6D) are presented. Conditional formatting of PD-L1 stains by five-rating icon sets of tumor cells and cells of TME (numbers in green font represent the percentage of positive cells in TME, including lymphocytes, macrophages, and around blood vessels in lighter orange rows) (Figure 6E) are presented. Semi-quantification of TILs is represented in the figure. * represents lymphovascular invasion, and ** represents poorly differentiated regions of the tumor. Each column represents data from tumor-adjacent normal tissue (D) and tumor tissue (E) from an individual patient. 

### 2.7. Plasma sPD-L1 and sPD-L2 Protein Expression Status from Peripheral Blood Samples of Patients with EC by ELISA

**Figure 7:**
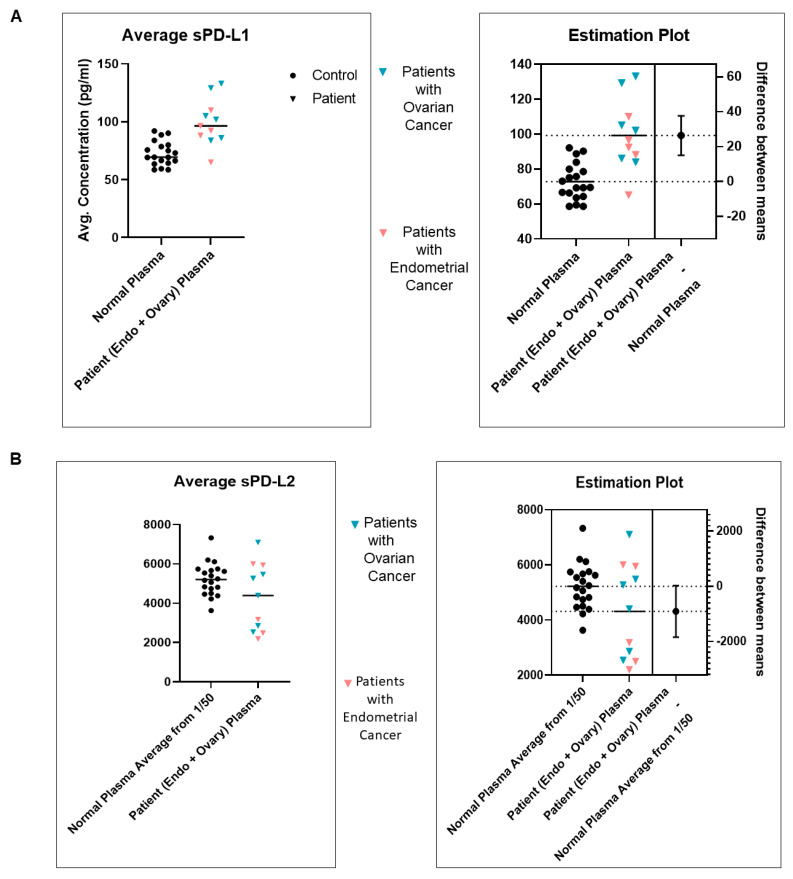

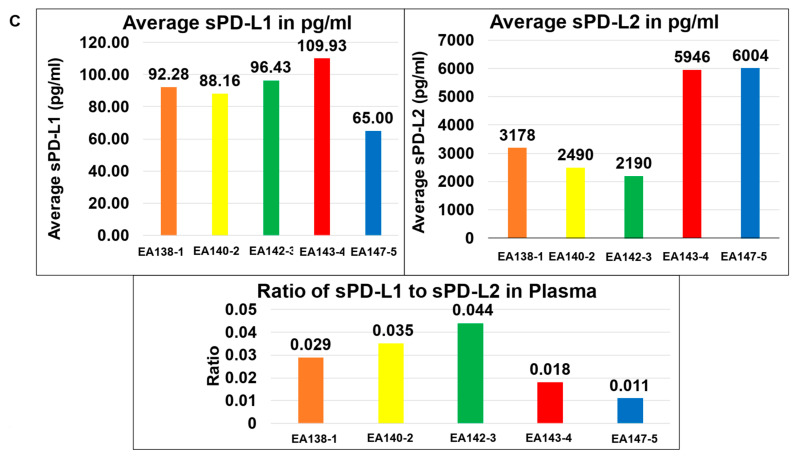
ELISA-based determination of plasma sPD-L1 and sPD-L2 from the blood of patients with endometrial cancers: Average sPD-L1 (**A**) and sPD-L2 (**B**) from the blood (plasma) of age-matched normal subjects (solid black circles) and patients with endometrial cancers (peach triangles) are presented. Data from patients with ovarian cancers were included as an internal control, as indicated by teal triangles. The difference between the means is presented in the estimation plot. The *p* value (unpaired *t*-test) is <0.0001 for sPD-L1 (n = 19 for normal subjects and n = 11 for cancer patients). The *p*-value (unpaired *t*-test) is <0.05 for sPD-L2 (n = 20 for normal subjects and n = 11 for cancer patients). The rightmost column in the estimation plot represents the difference between the means of the two groups (in pg/mL units). Hence in Figure 7A, the plot represents the difference between Patient (Endo + Ovary) Plasma and Normal Plasma, and it is a positive value as the expression in the Patient (Endo + Ovary) Plasma was higher than that in Normal Plasma PD-L1. In (**B**), the plot represents the difference between Patient (Endo + Ovary) Plasma and Normal Plasma Average from 1/50, and it is a negative value as the expression in the patient (Endo + Ovary) plasma was lower than that in Normal Plasma PD-L2 Average from 1/50. Plasma sPD-L1 and sPD-L2 expression by ELISA and their ratios in the blood of patients with endometrial cancers are plotted individually (**C**).

Standardization of sPD-L1 and sPD-L2 from plasma of blood from patients with EC was performed and compared to (1) 20 healthy age-matched subjects and (2) 6 patients with ovarian cancers (Figure 7A). The expression of plasma sPD-L1 from healthy age-matched controls was found to be lower than the levels observed in the blood of patients with EC. At the same time, the expressions of plasma sPD-L1 from patients with EC were found to be comparable to the levels observed in the blood of patients with ovarian cancers. The pattern was reversed in the case of PD-L2 (Figure 7B). The difference between the means is presented in the estimation plot. The *p* value (unpaired *t*-test) is <0.0001 for sPD-L1 (n = 19 for normal subjects and n = 11 for cancer patients). The *p*-value (unpaired *t*-test) is <0.05 for sPD-L2 (n = 20 for normal subjects and n = 11 for cancer patients). Plasma sPD-L1 and sPD-L2 expression by ELISA and their ratios in the blood of patients with EC are plotted individually (Figure 7C). 

### 2.8. Relationship between the Pathological Parameters and Tumor-TME Landscape of PD-L1/PD-L2 and PD-1 from Paired Samples of Tumor and Tumor-Adjacent Normal Tissues in EC

**Figure 8:**
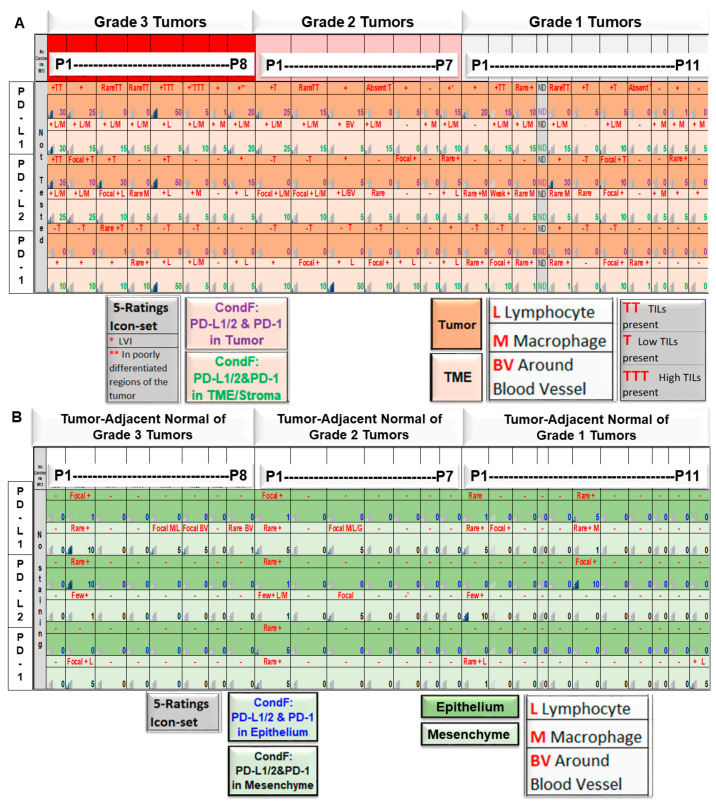
Conditional formatting by five-rating icon sets of paired T-TME (**A**) and N-SME (**B**) staining for PD-L1, PD-L2, and PD-1 by IHC on FFPE sections from Day0 that were used for sorting by grades 1, 2 and 3 of the disease. A. Conditional formatting of PD-L1, PD-L2, and PD-1 stains by five-rating icon sets of tumor cells (numbers in violet font represent Tumor Proportion Score, TPS (<%) in dark-orange filled rows) and cells of the TME (numbers in green font represent % of positive cells in the TME, including lymphocytes, macrophages, and around blood vessels, in lighter orange rows). Semi-quantification of TILs is represented in the figure. * represents lymphovascular invasion, and ** represents poorly differentiated regions of the tumor. (**B**). Conditional formatting of PD-L1, PD-L2, and PD-1 stains by five-rating icon sets of tumor-adjacent normal tissues (numbers in blue font represent positive epithelial cells (%) in dark green filled rows) and SME (numbers in black font represent % of positive cells in SME, including lymphocytes, macrophages, and around blood vessels, in lighter green rows).

Supplementary Table S1 shows pathological parameters, including histology, FIGO grade, stage, myometrial invasion, and LVI of tumor tissues from patients with EC, which were used in the study. Conditional formatting by five-rating icon sets of paired T-TME and N-SME staining for PD-L1, PD-L2, and PD-1 (by IHC on FFPE sections) from Day0 that were sorted by grade 1, 2, and 3 of the disease (Figure 8) are presented. Conditional formatting of PD-L1, PD-L2, and PD-1 stains by five-rating icon sets of tumor cells (numbers in violet font represent Tumor Proportion Score, TPS (<%) in dark-orange filled rows) and cells of TME (numbers in green font represents the percentage of positive cells in TME, including lymphocytes, macrophages, and around blood vessels, in lighter orange rows) are presented. Semi-quantification of TILs is represented in the figure (Figure 8A). Conditional formatting of PD-L1, PD-L2, and PD-1 stains by five-rating icon sets of tumor-adjacent normal tissues (numbers in blue font represent positive epithelial cells (%) in dark-green filled rows) and SME (numbers in black font represent the percentage of positive cells in SME, including lymphocytes, macrophages, and around blood vessels, in lighter green rows) are presented (Figure 8B). The expression levels of all proteins were significantly higher in both tumor as well as TME as compared to the tumor-adjacent normal epithelium and SME. The expression was higher in patients with grade 3 disease as compared to patients with grade 1 disease. On the contrary, the expression levels of all proteins were significantly lower in both epithelium as well as SME as compared to tumor and TME. Essentially no difference in the expression was observed between patients with different pathological parameters, including grades of the disease. Table 2 shows the correlation between the expression(s) of PD-L1, PD-L2, and PD-1 (by IHC on FFPE sections) and tumor grades. Tumor cells and cells within the TME/tumor stroma from endometrial tumor tissues are evaluated separately. The correlation between total “Tumor + TME Combined Scores” for each of PD-L1, PD-L2, and PD-1 and tumor grades 1, 2, and 3 are presented. Table 3 presents correlations between IHC expression of PD-L1 versus the percentage of myometrial invasion and IHC expression of PD-L1 versus the presence of TILs in tumor tissue from patients with EC.

Figure 6A–C presents the range of expression of PD-L1 in our CAFs derived from the tumor samples and tumor-adjacent normal tissues. We identified that the range of expression is wide, in both quantitative (from no expression in Figure 6C to diffuse to patchy high expressions in Figure 6A) and in subcellular patterns (from diffuse in Figure 6A to patchy high expressions in Figure 6C). We also recorded that the expression varied between NCAFs and TCAFs, TCAFs being higher in expression in the same patient (Figure 6C). As we demonstrated the wide range of expression, we wanted to test the expression from the perspective of the original tumor tissue and tumor-adjacent normal tissues from which these CAFs were derived. Figure 6D represents a heatmap expression of PD-L1 conditionally formatted from 15 patients. The figure shows that the source of PD-L1 positivity is predominantly CAF in tumor-adjacent normal tissues as the mesenchyme (blood vessels, lymphocytes, and macrophages) are rarely positive, and the epithelium is devoid of PD- L1 stain. In contrast, a heatmap expression of PD-L1 conditionally formatted from the tumor samples of 15 patients showed that in addition to higher levels of expression of PD-L1 in CAFs, both the tumor and the rest of the components of the TME expressed higher levels of expression of PD-L1 (Figure 6E), thus characterizing the PD-L1 expression in the tumor compartment. Delving into the combined score of the expression of PD-L1 in the tumor of these 15 patients, we identified that a patient-specific distribution of PD-L1, with a few patients expressing no-to-low levels (5%) of expression in the tumor and TME, as well as CAFs. Hence, we hypothesized that the pathological parameters in these patients might provide a clue for the differential expressions. We categorized the patient’s tumors according to their pathological grades (G1/G2/G3) and formatted the expression of PD-L1, PD-L2, and PD-1 in the tumor and TME compartments (Figure 8A). We observed a grade-dependent expression of PD-L1, PD-L2, and PD-1 in the tumor and TME compartments. In contrast, the tumor-adjacent normal tissues lack protein expression (Figure 8B). In testing the correlation between the expression(s) of PD-L1, PD-L2, and PD-1 and tumor grades, as expected, we found a significant positive correlation between the PD-L1 in the TME/tumor stroma (lymphocyte/macrophage/blood vessel) and grade 3 (when compared with grade 1) (Table 2). Interestingly, we observed a significant positive correlation between “Tumor + TME Combined Scores” and grades for PD-L1 and PD-L2. Our data indicated that the expression of PD-L1 in tumor samples from patients with grade 3 disease is contributed by tumor cells and TME compartments in endometrial cancers. Our data support the proposition that endometrial tumor cells, by virtue of their genomic alterations, augment the grade of the disease by dampening the immune surveillance of infiltrating T-cells via enhanced PD- L1-PD-1 reactions in the tumor. It is logical to argue that a higher expression of PD-L1 contributed by tumor cells may render TILs ineffective and exhausted, thus making the tumor more dependent on the PD-L1–PD-1 axis and conducive to being responsible for the disease’s higher grades (G3, more precisely). In support of our argument, we observed high TILs in five out of eight tumors with grade 3 (Figure 8A). Such a contention provides a stronger logical argument for the possibility of targeting the PD-L1–PD-1 axis of such tumors by ICI. We are currently working on more patient samples to provide conclusive data in this direction. 

### 2.9. MMR Status of PD-L1+ Tumor Cells (by IHC) Versus PD-L1+ TCAFs in Patients with EC 

Although PD-L1–PD-1 pathway status in the tumor tissues was historically considered the first biomarker designated for ICI treatment, DNA mismatch repair (MMR) status is a powerful tumor-agnostic biomarker for ICI [16]. In dMMR (deficient in MMR) EC, it has been associated with the success of ICI treatment with lenvatinib plus pembrolizumab and dostarlimab [17,18]. MMR-related genes are MLH1 (Chr. 3p22), MSH2 (Chr. 2p16.3), MSH6 (Chr. 2p16.3), and PMS2 (Chr. 7q22.2). Immunohistochemistry staining for PD-L1 was performed on all cases and scored in both the tumor and the peri-tumoral immune compartment (TME). Tumor staining was classified as positive when membranous (PD-L1) staining was present in ≥1% of tumor cells. Immune stromal staining was scored as positive when ≥5% of peritumoral and intratumoral immune cells (including lymphocytes and macrophages) showed reactivity. Supplementary Table S2 presents PD-L1 expression of tumor tissues and ex vivo tumor-tissue-derived CAFs in mismatch repair deficient endometrial carcinomas. Out of 19 tumors, 8 were found to be dMMR, while the remaining 11 were MMR-proficient. Whole sections of 19 formalin-fixed, paraffin-embedded endometrial tumor tissues obtained on the day of surgery were evaluated for MMR status, PD-L1 expression of the tumor tissue, and PD-L1 expression of the tumor tissue-derived primary CAFs. All eight dMMR patients had 100% abnormal PMS2, 100% normal MSH6, 62.5% abnormal MLH1, and 12.5% abnormal MSH2. All these patients had a higher probability of associated Lynch syndrome. We were interested in finding the relationship status between dMMR in patients with EC, PD-L1 positivity in tumors (by IHC), and PD-L1 positivity in TCAFs (by ICC). Out of 13 PD-L1+ TCAFs, we observed dMMR in 8 patients (61.5%); in contrast, out of 16 PD-L1+ tumors, we observed dMMR in 6 patients (37.5%). All eight dMMR patients had 100% PD-L1 positivity in TCAFs. Out of 17 cases where PD-L1 expression in primary TCAFs was carried out, 4 cases had no PD-L1 (<1% PD-L1 positivity) despite their PD-L1 positivity in tumor tissue, and all 4 of the tumors were MMR-proficient. Ten tumors (10/14, 71.4%) among the total 14 cases in which PD-L1 status was determined in both tumor tissue and TCAFs, demonstrated PD-L1 in TCAF. The 100% of tumors with PD-L1 expression in TCAF demonstrated concomitant tumoral PD-L1 expression. PD-L1 expression was higher in TCAFs with dMMR as compared with MMR-proficient tumors. However, within each category of PD-L1 expression, the ICI response was not correlated in our patient cohort. Both dMMR status and PD-L1 expression on the tumor cells are routinely used for ICI treatment in a variety of tumors, including endometrial tumors. It is worth mentioning that PD-L1 expression on the tumor cells is not a foolproof biomarker for the selection of ICI treatment in solid tumors. Our limited data indicating a positive relation between dMMR status and PD-L1 expression on TCAF may suggest that PD-L1 expression on TCAF may be an additive biomarker for ICI treatment in EC and raise provocative questions about the fact that there are certain immunologic parameters vary between localization of PD-L1 expression between TCAF and tumor cells. If yes, what is the mechanistic explanation for it? Answering this question will require further studies in a controlled experimental system. 

## 3. Discussion

**Figure 9:**
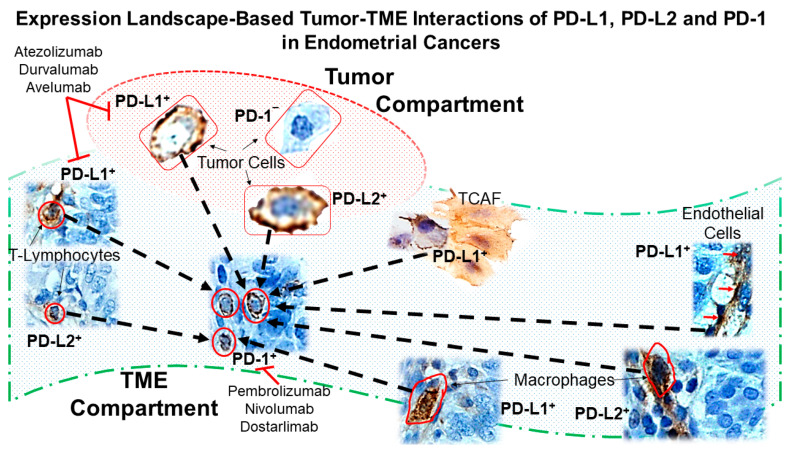
Diagrammatic presentation of tumor–TME interaction of PD-L1, PD-L2, and PD-1 in endometrial cancers based on the expression pattern of PD-L1, PD-L2, and PD-1. Based on the landscape of PD-L1, PD-L2, and PD-1 expression in the tumor compartment and TME compartment, PD-L1/2-PD-1-mediated immune checkpoint inhibition is proposed. Diagram presenting the expression pattern-based tumor–TME interaction of PD-L1, PD-L2, and PD-1 in endometrial cancers. The tumor-TME interaction based on the IHC expression pattern of PD-L1, PD-L2, and PD-1 in endometrial cancers demonstrated that PD-1-PD-L1/2 immune signaling could be blocked in the tumor stroma following the treatment with immune-checkpoint inhibitors (ICIs). The expression of PD-L1/2 in tumor cells, as well as macrophages and CAFs of the TME, are promising contributors to the inhibition of immune signaling. Pictures represent the expression of PD-L1/PD-L2 and PD-1 of tumor cells and cells of the tumor microenvironment, TME (lymphocytes, macrophages, and cancer-associated fibroblasts designated as TCAF) of FFPE from patients’ tumor tissue samples. The red square is a tumor cell; the red circle is a lymphocyte; the red freeform is a macrophage. The red arrow indicates PD-L1-positive endothelial cells. The dashed arrow represents the possible interaction between PD-L1/PD-L2 with their cognate receptor, PD-1.

We examined the possibility of an immune checkpoint inhibition involving the PD-1/PD-L1-based bipartite landscape in EC. The bipartite landscape represented tumor cells, tumor immune cells, and tumor CAFs, as compared with the stromal immune microenvironment of the paired tumor-adjacent normal tissues. Following the above-mentioned plan of the study, a summary of data (Figure 1, Figure 2, Figure 3, Figure 4, Figure 5, Figure 6, Figure 7, Figure 8 and Figure 9) generated from tumor and blood samples obtained from patients with endometrial cancers is presented in Table 4.

Viewing the evolving ecosystems of cancers as being composed of cells of heterogeneous types undergoing frequency-dependent selection, the tumor–TME model has been mathematically framed as an evolutionary game by Benjamin Wölfl et al. [19] in order to conceptualize and analyze biological interactions where tumor cells’ fitness not only depends on one’s own traits but also on the traits of cells of the TME. Interactions between cancer cells and/or interactions between cancer cells and the TME through the Lotka–Volterra competition equations and their extensions (termed the “Deadlock game” and “Leader game”), have been proposed based on the presence or absence of drug and/or cancer-associated fibroblasts. In fact, studies by Kaznatcheev et al. [20] demonstrated that cancer-associated fibroblasts qualitatively switch the type of game being played by the in-vitro population from Leader to Deadlock, providing empirical confirmation of a central theoretical postulate of evolutionary game theory in oncology in the non-small cell lung cancer model. In their system, an untreated tumor was similar to DMSO + CAF and thus followed the Leader game. Treating with Alectinib (move to Alectinib + CAF) or eliminating CAFs through a stromal-directed therapy (move to DMSO) moved the game into a Deadlock game. As a component of the TME, the CAF is a *partner in crime* in EC [8]. Endometrial CAFs are known to promote cancer growth by activating tumorigenic pathways involving several tumor cell phenotypes [21,22,23,24]. PD-L1 expression profiles differ between molecular subclasses, histologic subtypes, and disease stages of EC, and PD-L1 is a biomarker for predicting the response to PD-1/PD-L1 immunotherapy [14]. Our finding of a PD-L1-dominated tumor-CAF-immune landscape provides the groundwork for establishing crosstalk within the TME in EC.

The landscape of PD-L1, PD-L2, and PD-1 in the endometrial tumor compartment and endometrial TME compartment can lead to PD-L1/2-PD-1-mediated immune checkpoint inhibition, as represented in Figure 9. Schematic presentation of the tumor–TME interaction of PD-L1, PD-L2, and PD-1 in EC is based on the expression pattern of PD-L1, PD-L2, and PD-1. The tumor–TME interaction based on the IHC expression pattern of PD-L1, PD-L2, and PD-1 in EC demonstrated that PD-1-PD-L1/2 immune signaling could be blocked in the tumor stroma. The expression of PD-L1/2 in tumor cells, as well as macrophages and CAFs of the TME, are promising contributors to the inhibition of immune signaling. A PD-L1/2-PD-1-mediated immune checkpoint inhibition could be proposed to occur involving cells of (A) tumor and TME compartments, as well as (B) within different component cells of the TME (T-cells, CAFs, and macrophages) in EC. Tumor cells express PD-L1 and PD-L2, which are ligands for PD-1 receptors. PD-1 receptors are present in the infiltrating T-lymphocytes of TME. PD-L1 and PD-L2 are also expressed on (1) T-lymphocytes, (2) CD68+ and CD163+ macrophages, and (3) SMA+/FAP+/S100A4+/TE-7+/CD90+/EpCAM-/CK8,18-/CD31- CAFs of the TME. Thus, (1) PD-L1 and PD-L2 expressing tumor cells, (2) PD-L1 and PD-L2 expressing infiltrating T-cells of the TME, (3) PD-L1 expressing CAFs of the TME, and (4) PD-L1 and PD-L2 expressing macrophages of the TME can initiate PD-L1/L2-mediated immune checkpoint inhibition by binding with PD-1 receptors expressed on CD3+/CD4+/CD8+ T-lymphocytes in the TME. 

PD-L1 and CD4 are reported to be independent prognostic factors for overall survival in EC [25], and the synergistic effect of inhibition of PD-1/PD-L1 checkpoint and PARP has been reported in recurrent or metastatic endometrial cancer [26]. Studying the PD-L1 and mismatch repair status in uterine carcinosarcomas, Jenkins et al. reported that PD-L1 might be additive to MMR testing as a predictive biomarker for checkpoint inhibitors [27]. Due to the presence of immune dysregulation in EC, immune checkpoint blockade therapy has been approved [28]. FDA granted accelerated approval of lenvatinib and pembrolizumab combination therapy in 2019 for the treatment of advanced endometrial cancers with proficient MMR. Currently, PD-L1, along with TMB and dMMR, is recommended by the FDA as a positive predictive biomarker for ICI treatment. Our data raise a thought-provoking question on the possibility of using the differential expression of the PD-L1/PD-L2/PD-1 on different cells during the treatment course and whether the expression can be used to predict the treatment outcome using ICI. Such a study would have addressed the futuristic aspect of whether PD-L1/PD-L2/PD-1 expression on different cells can be used as a prognostic marker in EC. Keeping in mind the value of the differential expression of the PD-L1/PD-L2/PD-1 on different cells on the treatment course, we standardized the soluble PD-L1/PD-L2/PD-1 expressions in the plasma of patients as presented in this study with the age-matched normal subjects as controls. A future study is warranted to test the expression of these proteins from the longitudinal blood draw during the course of the treatment. 

With the success of trials and the FDA approval of a number of ICI drugs in different solid tumors, including EC [16], driven by the knowledge that PD-L1 staining is one of the reliable predictive biomarkers [10,29], the contribution of CAF-mediated signals in influencing the tumor immune environment is undeniable [30]. Hence, studies on the landscape of PD-L1–PD-1 signaling have gained momentum in recent years in several solid tumors, including EC [31,32,33,34]. We present a detailed landscape of PD-L1 in tumor tissue, TME, tumor-adjacent epithelial tissue, SME, and cancer-associated fibroblast (TCAFs and NCAFs) that likely contribute to immune escape and tumor aggravation. It would have been interesting to find the correlation between PD-L1 expression in CAFs and patient survival or treatment efficacy with immune checkpoint inhibitors (ICIs) since it would provide valuable insights into the clinical relevance of the findings beyond the expression patterns.

Our PD-L1 expression of tumor tissue and tumor-tissue-derived CAFs in mismatch repair deficient endometrial carcinomas shed new light on the relationship between CAF PD-L1 and dMMR. Predictors of non-response in mismatch repair deficiency in epithelial cancers are poorly understood [12,16]. Both tumoral and immune cell expression of PD-L1 was statistically significantly associated with mismatch repair deficient tumors in EC [35]. We evaluated PD-L1 expression in our cohort of EC by mismatch repair status and PD-L1 expression in tumor-derived CAFs. Tumors demonstrating CAF+ PD-L1 expression were more likely to express dMMR. CAF+ PD-L1 expression could be better associated with a potential predictive additive biomarker for non-response to immune-checkpoint inhibition in dMMR tumors. CAF+ PD-L1 expression status could be considered as a translational endpoint in future clinical trials of immune checkpoint inhibition in endometrial cancer. Studies have shown that MMR deficiency is a better predictor of response to PD-1/PD-L1 inhibitor therapy than tumor grade in EC, and immune PD-L1 expression was observed in 100% of dMMR cases as compared to 66% of MMR-proficient cases [36]. Our results are in line with a previously documented association between MMR deficiency and PD-L1 expression [37]. Data indicated a novel association between dMMR and CAF PD-L1 expression, a possible potential additive biomarker of immune checkpoint inhibitor response in endometrial carcinoma, which remains to be confirmed in the future, to be tested and proven in larger cohorts, with longer follow-up data.

Our study has certain limitations. Although our total number of patients exceeded 100 in number, we had a low number of cases with ex vivo culture of patient-derived TCAFs to answer certain questions. Secondly, most of our patients were of endometrial endometrioid carcinoma histology with a clear skewness to grades 1 and 2, as we received consent from consecutive patients admitted to our Avera Cancer Institute. Third is the short follow-up time since surgery of less than 18 months. Hence, the outcome data for this ongoing study had not matured enough for analysis. In this study, we embarked on determining the landscape of the expression of PD-L1/PD-1 in endometrial tumor samples (tumor compartment and TME compartment) and whether this expression correlates to any of the established pathological parameters (grade, stage, LVI, myometrial invasion, and others). In testing the correlation with several pathological parameters, we observed a significant correlation between PD-L1 in grade 3 and PD-L1 in grade 1, which showed that the expression of PD-L1 protein is higher in patients with grade 3 as compared to the expression of PD-L1 protein in patients with grade 1. Although the relationship between PD-L1/PD-1 and tumor cell grade presented in our study is only a correlative association, our data may provide the groundwork for the answer to a provocative question of whether there is a direct functionalcause–effect relationship between PD-L1 expression and grade in endometrial cancers. We are currently studying the clinical relevance of our observed correlation to find out the possibility of pharmacological intervention. 

In summary, the expression of immune-checkpoint proteins PD-L1 and PD-L2 is primarily tumor-driven and associated with invasive endometrioid and serous adenocarcinoma, and more commonly with myometrial invasion. The expression of PD-L1 and PD-L2 is distributed in both tumor cells and cells of the TME and SME, while PD-1 expression is exclusively T-cell specific within the TME and SME. PD-L1 expression dominates in the lymphocytes, CAFs, and macrophages within the TME and is correlated to the high grade. Our data may provide evidence for a futuristic thought that PD-L1-positive CAFs might play a role in the functionality of CD8-positive cytotoxic T-cells. Hence PD-L1 expression in CAFs should be considered a contributing factor when evaluating the immune environment of the disease, T-cell exhaustion, and, therefore, tumor progression in EC.

## 4. Methods and Materials

To map the landscape of PD-L1/L2 and PD-1 in patients with endometrial cancers, we used tumors as well as peripheral blood. We obtained data from three types of samples from each patient with endometrial cancers: (1) resected tumor tissues, (2) resected tumor-adjacent normal tissues, and (3) peripheral blood on the day of the surgery. Resected tumor tissues were used for (1) testing the expression of PD-L1/L2 and PD-1 in tumor cells and cells of the TME following IHC from FFPE sections and (2) ex vivo primary culture of cancer-associated fibroblasts (TCAFs). Resected tumor-adjacent normal tissues were used for (1) testing the expression of PD-L1/L2 and PD-1 in tumor-adjacent normal epithelial cells and cells of the SME following IHC from FFPE section and (2) ex vivo primary culture of cancer-associated fibroblasts (NCAFs). Expressions of PD-L1/L2 were determined from the TCAFs and NCAFs by qRT-PCR, flow cytometry, and ICC. We determined soluble PD-L1/L2 from patients’ blood samples by ELISA compared to blood samples from the age-matched healthy subjects.

### 4.1. Patient Consent and Enrollment

Informed consent(s) were obtained from 149 patients with endometrial and ovarian cancers enrolled for the IRB-approved research protocol (*Protocol Number Study:2017.053-100399_ExVivo001*). Over 116 surgically resected tumors and tumor-associated normal tissues were obtained from consented (unselected consecutive) patients with EC at the Avera Cancer Institute between 16 March and 21 September.

### 4.2. Tissue and Blood Collection

Resected fresh tumor tissue(s) and tumor-adjacent normal tissue(s) were obtained from the pathology department. Samples were collected in DMEM/F-12 + Glutamax 500 mL (Base) supplemented with HyClone Penicillin-Streptomycin 100 × 100 mL (1%). Surgically resected tumor and tumor-associated normal tissues were obtained per the pathologist’s recommendation, and the pathology reports for the respective patients were included in the study. Blood was collected in cell-save tubes (CellSave Preservative Tubes, Menarini Silicon Biosystems, BrynAthyn, PA, USA catalog #7900005), and plasma was stored at −80 °C for sPD-L1, sPD-L2, and sPD-1 determination by ELISA (Abcam, Boston, MA, USA; Human PD-L1 ELISA kit, Product #ab277712; Abcam Human PD-L2 ELISA kit, Product #ab231928; Abcam Human PD-1 ELISA kit, Product #ab252360) as per the manufacturer’s protocol. For PD-L1/PD-1, plasma samples were used undiluted (50 μL sample) and diluted 1:1 in a diluent buffer. For PD-L2, plasma samples were first diluted at 1:10. From this 1:10 dilution, final dilutions of 1:25 and 1:50 were made for the assay. Samples were run in triplicate as desired/necessary, parallel to the standard curves for each batch.

### 4.3. IHC Expression of PD-L1, PD-L2, and PD-1 in FFPE Sections from Tumor and Tumor-Adjacent Normal Tissues

IHC expression of PD-L1, PD-L2, and PD-1 was carried out on FFPE sections from tumor and tumor-adjacent normal tissues. IHC expression of PD-L1 (22C3; Dako # M3653), PD-L2 (D7U8C: Cell signaling # 82723), and PD-1 (ABCAM # ab137132) was performed on resected tissues which were processed within an hour of surgery to preserve different types of cells including tumor cells, and cells of the TME and SME. The IHC detection kits were procured from Dako (Envision+ Dual-link system-HRP (DAB+), code K4065; Envision GI2 Doublestain system, Rabbit/Mouse (DAB+/Permanent Red), code K5361), and Abcam (ab210059 DoubleStain IHC Kit: M&R on human tissue (DAB and AP/Red). The validation of the protein expression was carried out in FFPEs of tonsil and tumor tissues. A board-certified pathologist evaluated the staining intensity and distribution pattern of expression of proteins by applying the standard scoring protocol and guidelines using a standard scoring system of TPS (1% cut-off value) and CPS (1 cut-off value) [10].

### 4.4. Primary Culture of Cancer-Associated Fibroblasts from Tumor and Tumor-Adjacent Normal Tissues

Since fibroblasts are the most abundant cells of TME, the mesenchymal/stromal/TME expression of the PD-L1 was separately and parallelly evaluated in the CAFs from surgically resected tumor tissues (tumor CAF, TCAF) and tumor-adjacent normal tissues (tumor-adjacent normal CAF, NCAF). For this purpose, we established primary cultures of CAFs from endometrial tumors and tumor-adjacent normal tissues. Primary cultures were set up from surgical samples within an hour of resection using a feeder plate technique in media (DMEM/F-12 + Glutamax, Fisher, Product #10-565-042; HyClone Fetal Bovine Serum 0.1 uM Sterile Filtered, Cytiva Life Sciences, Marlborough, MA, USA; Product #SH30910.03; HyClone Penicillin-Streptomycin 100×, Cytiva Life Sciences, Product #SV30010; Bovine Serum Albumin Solution, Sigma, Burlington, MA, USA; Product #A8412; HyClone HEPES Buffer Liquid, Cytiva Life Sciences, Product #SH30237.01). Cultured primary CAFs were assessed (by flow cytometry, ICC, and qRT-PCR) for expression of the set of CAF markers, SMA, FAP, CD90, S100A4, TE-7, and negative expression of epithelial tumor markers, including EpCAM as well as CK8, 18 and negative expression for the endothelial marker, CD31. The % of the expression of epithelial (EpCAM), fibroblast (SMA, S100A4, FAP, and CD90), and endothelial (CD31) marker proteins were tested using flow cytometry. The passages were maintained until the cells ceased to grow and tested for senescence by beta-galactosidase staining. Parallel evaluation of PD-L1 in the multiple passages of cultured NCAFs and TCAFs by the qRT-PCR, flow cytometry, and ICC was carried out, as mentioned elsewhere [15].

### 4.5. Flow Cytometric Expression of PD-L1 in Primary Culture of CAF from Tumor and Tumor-Adjacent Normal Tissues

We tested the expression of PD-L1 protein by flow cytometry (BD C6 Accuri). NCAFs and TCAFs were trypsin released and rinsed in FACS buffer (Phenol red-free RPMI with 1% FBS). Cells were resuspended in 100 μL FACS buffer, and the corresponding antibody was added and incubated at 4 °C in the dark for 20 min. (PD-L1-APC or isotype control-APC Milteyni Biotec, Gaithersburg, MD, USA). Cells were rinsed with 2 mL FACS buffer and then resuspended in 300 μL FACS buffer for flow cytometry analysis. Cells were analyzed on a BD C6 Accuri, as mentioned earlier [15].

### 4.6. Relative Gene Expression of PD-L1 and PD-L2 in Primary Culture of CAF from Tumor and Tumor-Adjacent Normal Tissues by qRT-PCR 

The abundance of gene-specific transcripts (mRNAs) for PD-L1 and PD-L2 in CAFs of both tumors and tumor-adjacent normal samples were examined by 2-step qRT-PCR. First, total RNA was extracted (RNeasy Mini Kit, Qiagen, Germantown, MD, USA; Product#74106), followed by cDNA conversion (iScript Reverse Transcription Supermix, Bio-Rad, Hercules, CA, USA; Product #1708841) and quantitation on a Roche Light Cycler 96 qPCR system. 

All primers are procured from Integrated DNA Technologies, and sequences listed 5′-3′:

○ PD-L1 Forward: ACC TAC TGG CAT TTG CTG AAC G;
○ PD-L1 Reverse: ATA GAC AAT TAG TGC AGC CAG GT;
○ PD-L2 Forward: TGG AAT TGC AGC TTC ACC AGA;
○ PD-L2 Reverse: TGG CTG TTA TTG CTC CAA GGT;
○ GAPDH Forward: TGC ACC ACC AAC TGC TTA GC;
○ GAPDH Reverse: GGC ATG GAC TGT GGT CAT GAG.

### 4.7. ICC Expression of SMA, S100A4, TE-7, EpCAM, CK 8, 18, and PD-L1 in Primary Culture of CAFs from Tumor and Tumor-Adjacent Normal Tissues

We characterized patient-derived CAFs by testing the expression of SMA (Actin, Smooth Muscle, 1A4; Cell Marque catalog # 202M-94), S100A4 (Recombinant Anti-S100A4 antibody; Abcam catalog # ab124805), TE-7 (Fibroblasts Antibody, TE-7; NOVUS Biologicals, LLC, Centennial, CO, USA; catalog # NBP2-50082), and negative expression of epithelial tumor markers including EpCAM (Ep-CAM/Epithelial Specific Antigen, Ber-EP4; Cell Marque, Rocklin, CA, USA; catalog # 248M-94) as well as CK 8, 18 (Cytokeratin 8 and 18; B22.1 and B23.1 from Cell Marque; catalog # 818M-94). Human uterine fibroblasts (HUF) and NCI-H441 tumor cell lines were used for validation. The percentage of PD-L1 (PD-L1 [Clone 22C3]; Agilent-Dako, Santa Clara, CA, USA; catalog # M365329-1) expression was tested in well-characterized CAFs from both tumors and tumor-adjacent normal samples. The IHC detection kit was procured from Dako (Envision+ Dual-link system-HRP (DAB+), code K4065).

## 5. Patent Status

A portion of the study presented in the MS is part of a patent application (United States Patent and Trademark Office; Application number 16/875,910.

## Figures and Tables

**Table 1:** Comparing the significance of IHC expressions of PD-L1, PD-L2, and PD-1 in (1) epithelium vs. SME, (2) tumor vs. TME, (3) epithelium vs. tumor, and (4) TME vs. SME in tumor and tumor-adjacent normal tissues from patients with endometrial cancers. *p* values lower than 0.05 are considered significant.

**PD-L1**				
T-test: Type 2, and Two Tail; n = 26 patients in each group	Difference in PD-L1 expression: Epithelium vs. SME	Difference in PD-L1 expression: Tumor vs. TME	Difference in PD-L1 expression: Epithelium vs. Tumor	Difference in PD-L1 expression: TME vs. SME
*p* Value	*p* = 0.0496	*p* = 0.936	*p* = 0.00015	*p* = 0.00000027
**PD-L2**				
T-test: Type 2, and Two Tail; n = 26 patients in each group	Difference in PD-L2 expression: Epithelium vs. SME	Difference in PD-L2 expression: Tumor vs. TME	Difference in PD-L2 expression: Epithelium vs. Tumor	Difference in PD-L2 expression: TME vs. SME
*p* Value	*p* = 0.82	*p* = 0.5971	*p* = 0.0110	*p* = 0.0000069
**PD-1**				
T-test: Type 2, and Two Tail; n = 26 patients in each group	Difference in PD-1 expression: Epithelium vs. SME	Difference in PD-1 expression: Tumor vs. TME	Difference in PD-1 expression: Epithelium vs. Tumor	Difference in PD-1 expression: TME vs. SME
*p* Value	*p* = 0.261	*p* = 0.003045	*p* = 0.03486	*p* = 0.0023

**Table 2:** Correlation between the expression(s) of PD-L1, PD-L2, and PD-1 (by IHC on FFPE sections) and tumor grades; tumor cells and cells within TME/tumor stroma from endometrial tumor tissues are evaluated separately. Correlation between total “Tumor + TME Combined Scores” for each of PD-L1, PD-L2, and PD-1 and tumor grades 1, 2, and 3 are presented.

**Proteins**	**Correlation between Expression(s) of PD-L1, PD-L2 and PD-1:** **Grade 3 vs. Grade 1**					
	**PD-L1**		**PD-L2**		**PD-1**	
**Expression Site** **of the ** **Proteins**	**Expression in Tumor** **Cells**	**Expression in TME/** **Tumor Stroma** **(Lymphocyte/Macrophage/Blood Vessel)**	**Expression in Tumor** **Cells**	**Expression in TME/** **Tumor Stroma (Lymphocyte/Macrophage/Blood Vessel)**	**Expression in Tumor** **Cells**	**Expression in TME/** **Tumor Stroma (Lymphocyte/Macrophage/Blood Vessel)**
r	0.3964	0.4745	0.4294	0.3961	−0.19	0.444
R^2^	0.1571	0.2252	0.1844	0.1569	0.03609	0.1971
*p* (Two-tailed test)	0.1034	0.0466	0.0753	0.1037	0.4502	0.0649
**Significant ** **(alpha = 0.05)**	Not Significant	**Significant**	Not Significant	Not Significant	Not Significant	Not Significant
# of Pairs	18	18	18	18	18	18
Proteins	**Correlation between expression(s) of PD-L1, PD-L2 and PD-1:** **Grade 2 vs. Grade 1**					
	**PD-L1**		**PD-L2**		**PD-1**	
Expression site of the proteins	**Expression in Tumor** **Cells**	**Expression in TME/** **Tumor Stroma ** **(Lymphocyte/Macrophage/Blood Vessel)**	**Expression in Tumor** **Cells**	**Expression in TME/** **Tumor Stroma (Lymphocyte/Macrophage/Blood Vessel)**	**Expression in Tumor** **Cells**	**Expression in TME/** **Tumor Stroma (Lymphocyte/Macrophage/Blood Vessel)**
r	0.3141	0.2148	−0.1605	0.077	−0.0548	0.503
R^2^	0.0987	0.0461	0.0258	0.0059	0.003	0.253
*p* (Two-tailed)	0.2195	0.4077	0.5383	0.7689	0.8344	0.0396
**Significant ** **(alpha = 0.05)**	Not Significant	Not Significant	Not Significant	Not Significant	Not Significant	**Significant**
# Pairs	17	17	17	17	17	17
Proteins	**Correlation between “Tumor + TME Combined Scores” for PD-L1, PD-L2 and PD-1:** **Tumor Grades 1, 2 and 3**					
	**PD-L1**		**PD-L2**		**PD-1**	
Expression site of the proteins	**Expression in Tumor** **Cells and Cells of TME**		**Expression in Tumor** **Cells and Cells of TME**		**Expression in Tumor** **Cells and Cells of TME**	
r	0.4609		0.4573		0.2918	
R^2^	0.2124		0.2091		0.08513	
*p* (Two-tailed test)	0.0178		0.0188		0.1481	
**Significant ** **(alpha = 0.05)**	**Significant**		**Significant**		Not Significant	
# Pairs	26		26		26	

**Table 3:** Correlations between IHC expression of PDL1 versus percentage of myometrial invasion and IHC expression of PDL1 versus presence of TILs in tumor tissue from patients with endometrial cancers.

Site	Correlations	R-Value	R^2^ Value	*p* Value	Significance	# of Patients (N)
**TUMOR**	Tumor PD-L1 by IHC vs. Percentage of Myometrial Invasion	0.2085	0.04345	0.3068	Not Significant	26
**TME**	TME PD-L1 by IHC vs. Percentage of Myometrial Invasion	0.4524	0.2047	0.0203	**Significant**	26
**TUMOR + TME**	Tumor + TME combined PD-L1 by IHC vs. Percentage of Myometrial Invasion	0.3688	0.1360	0.0637	Not Significant	26
	Tumor + TME combined PD-L1 by IHC Cutoff = 15 vs. Percentage of Myometrial Invasion	0.3665	0.1343	0.0656	Not Significant	26
**TUMOR**	Tumor PD-L1 by IHC vs. Presence of TILs	0.1560	0.02433	0.4467	Not Significant	26
**TME**	TME PD-L1 by IHC vs. Presence of TILs	0.4901	0.2402	0.0110	**Significant**	26
**TUMOR + TME**	Tumor + TME combined PD-L1 by IHC vs. Presence of TILs	0.3480	0.1211	0.0815	Not Significant	26
	Tumor + TME combined PD-L1 by IHC Cutoff = 15 vs. Presence of TILs	0.4501	0.2025	0.0211	**Significant**	26

**Table 4:** A summary of data generated from tumor and blood samples obtained from patients with endometrial cancers.

Figure #	Sub-Figure	Data Presented	Summary of Data
**1**	A	Overall Plan of the Study	Data generated from patients’ tumor tissue, tumor-adjacent normal tissue, and blood
	B	PD-L1 status of tumor cells	PD-L1 +ve Tumor cells
	C	PD-L2 status of tumor cells	PD-L2 +ve Tumor cells
	D	PD-1 status of tumor cells	PD-1 −ve Tumor cells
	E	PD-L1 status of cells of TME	PD-L1 +ve Lymphocytes
	F	PD-L1 status of cells of TME	PD-L1 +ve Macrophage
	G	PD-L2 status of cells of TME	PD-L2 −ve Tumor Cells and Lymphocyte
	H	PD-L2 status of cells of TME	PD-L2 +ve Macrophage and Lymphocyte
	I	PD-1 status of cells of TME	PD-1 +ve Lymphocytes
**2**	A	PD-L1 status of cells of SME in tumor-adjacent normal tissue	PD-L1 +ve Lymphocyte
	B	PD-L2 +ve epithelium of tumor-adjacent normal tissue	PD-L2 −ve normal Epithelium and Macrophage
	C	PD-1 status of tumor-adjacent normal tissue	PD-1 −ve Epithelium and PD-1 +ve Lymphocytes
	D	PD-L1 +ve SME of tumor-adjacent normal tissue	PD-L1 +ve Lymphocyte and PD-L1 −ve Epithelium
	E	PD-L2 +ve SME of tumor-adjacent normal tissue	PD-L2 +ve Mesenchyme
	F	PD-1 +ve SME of tumor-adjacent normal tissue	PD-1 +ve Lymphocyte and PD-1 −ve Epithelium
**3**	A	Flowcytometric expression of positive and negative CAF markers in NCAF and TCAF	Positive expression of FAP-A, SMA, and negative expression of S100A4, CD31, EpCAM
	B	Flowcytometric expression of positive and negative CAF markers in NCAF and TCAF	Positive expression of FAP-A, SMA, S100A4, CD90 and negative expression of CD31, EpCAM
	C	Flowcytometric expression of positive and negative CAF markers in NCAF and TCAF	Positive expression of FAP-A, SMA, CD90 and negative expression of CD31, EpCAM, S100A4
	D	Percentage of expression of epithelial (EpCAM), fibroblast (SMA, S100A4, FAP, and CD90), and endothelial (CD31) marker proteins by flow cytometry	Both TCAFs and NCAFs are positive for SMA, CD90, and FAP-A and negative for CD31 and EpCAM. S100A4 is differentially expressed.
	E	Expression of CK 8, 18, and EpCAM in NCAF from passages 1, 2, and 3	Negative expression of CK 8, 18, and EpCAM in NCAF
	F	Expression of CK 8, 18, and EpCAM in TCAF from passages 1, 2, and 3	Negative expression of CK 8, 18, and EpCAM in TCAF
	G	Expression of SMA, S100A4, TE-7, and PD-L1 in NCAF from passages 1, 2, and 3	Positive expression of SMA, TE-7 and negative expression of S100A4, PD-L1 in NCAF
	H	Expression of SMA, S100A4, TE-7, and PD-L1 in TCAF from passages 1, 2, and 3	Positive expression of SMA, TE-7 and negative expression of S100A4, PD-L1 in TCAF
	I	Percentage of expression of epithelial (EpCAM and CK 8,18) and fibroblast (SMA, S100A4, and TE-7) marker proteins by ICC	Both TCAFs and NCAFs are positive for SMA and TE-7 and negative for CK 8, 18, and EpCAM. S100A4 is differentially expressed.
**4**	A	Relative Ratio of PD-L1 by qRT-PCR	TCAFs tend to express higher levels of PD-L1 mRNA than NCAFs
	B	Relative Ratio of PD-L2 by qRT-PCR	TCAFs tend to express higher levels of PD-L2 mRNA than NCAFs
	C	Heatmap of PD-L1 and PD-L2 mRNA expressions in different patients	Ratios of PD-L1 and PD-L2 mRNA expressions in TCAFs/NCAFs pairs are patient-specific.
**5**	A	Flow cytometric expression of PD-L1 in NCAFs and TCAFs pairs	PD-L1 expression tends to be higher in TCAFs compared to TCAFs.
	B		
	C		
**6**	A	ICC expression of PD-L1 in TCAF-NCAF pair	PD-L1 is expressed at a comparable level in both NCAFs and TCAFs
	B		PD-L1 expressed in NCAFs is higher than TCAFs
	C		PD-L1 expressed in TCAFs is higher than NCAFs
	D	Conditional formatting of the IHC (FFPE of tumor-adjacent normal epithelium/mesenchyme) vs. ICC (NCAFs) expression of PD-L1 in normal epithelium and mesenchyme in the same patient	The epithelium is rarely positive for PD-L1 compared to NCAFs in the same patient.
	E	Conditional formatting of the IHC (FFPE of tumor/TME) vs. ICC (TCAFs) expression of PD-L1 in tumor cells and TME in the same patient	Expression of PD-L1 in TCAFs is higher than tumor/TME in the same patient.
**7**	A	Standardization of sPD-L1 from plasma of blood from patients with EC as compared to the normal subjects	sPD-L1 from the plasma of blood from patients with EC is higher than sPD-L1 from the blood of healthy normal subjects
	B	Standardization of sPD-L2 from plasma of blood from patients with EC as compared to the normal subjects	sPD-L2 from the plasma of blood from patients with EC is comparable to sPD-L2 from the blood of healthy normal subjects
	C	The ratio of sPD-L1 to sPD-L2 in the Plasma of patients with EC	The average sPD-L1 is lower than the average sPD-L2 in the Plasma of patients with EC.
**8**	A	Expression pattern of PD-L1, PD-L2, and PD-1 in tumor cells/TME of patients with grade 1, 2, and 3 diseases as obtained from tumor samples	Expression of PD-L1 is correlated to the highest grade of the disease.
	B	Expression patterns of PD-L1, PD-L2, and PD-1 in epithelium/SME of patients with grade 1, 2, and 3 diseases were obtained from tumor-adjacent normal tissue samples.	No correlation was observed between the expression of PD-L1 with the grade of the disease.
**9**		Diagram presenting the expression pattern-based tumor–TME interaction of PD-L1, PD-L2, and PD-1 in endometrial cancers.	Expression landscape-based tumor–TME interactions of PD-L1, PD-L2 and PD-1 in endometrial cancers

## Data Availability

The study was translational/experimental in nature. No public data was used. All experimental data are presented in the figures or as supplementary data.

## Supplementary Materials

The following supporting information can be downloaded at: https://www.mdpi.com/article/10.3390/ijms241311079/s1.
