# Supplementary material for: Tumor-TME Bipartite Landscape of PD-1/PD-L1 in Endometrial Cancers
Source: Int J Mol Sci. 2023 Jul 4;24(13):11079. doi: 10.3390/ijms241311079 (PMC10342322; doi:10.3390/ijms241311079)
Supplement: Supplementary file 1 [file ijms-24-11079-s001.zip › ijms-2375504-supplementary.pdf]

Supplementary Table S1: Pathological Parameters of Tumor Tissues from Patients with Endometrial Cancers

Tumor Tissue	Pathological Parameters				
De-identified Patient #	Histology	FIGO Grade	Stage	Myo-metrial Invasion (%)	LVI
A99	Endometrioid adenocarcinoma	3	IB	95	Absent
A101	Endometrioid adenocarcinoma	2	I	11	Absent
A102	Endometrioid adenocarcinoma	1	IA	29	Absent
A103	Endometrioid adenocarcinoma	3	IA	43	Absent
A104	Endometrioid adenocarcinoma	1	IA	34	Present
A107	Endometrioid adenocarcinoma	1	IA	34	Absent
A108	Endometrioid adenocarcinoma	1	IA	13	Absent
A111	Endometrioid adenocarcinoma	3	IA	37	Absent
A112	Endometrioid adenocarcinoma	1	IA	35	Absent
A114	Endometrioid adenocarcinoma	3	IB	90	Absent
A115	Endometrioid adenocarcinoma	2	IA	15	Absent
A116	Endometrioid adenocarcinoma	2	IA	32	Absent
A117	Endometrioid adenocarcinoma	2	IA	8	Absent
A118	High-grade serous adenocarcinoma	3	IIIC2	46	Present

A119	Endometrial carcinoma, NOS	**1**	**IA**	**0**	**Absent**
A124	Endometrioid adenocarcinoma	**1**	**IA**	**38**	**Absent**
A125	Endometrioid adenocarcinoma, noninvasive	**1**	**IA**	**0**	**Absent**
A129	High-grade serous adenocarcinoma	**3**	**IIIC2**	**87**	**Present**
A131	Endometrial cancer	**1**	**IVB**	**0**	**Present**
A134	Endometrioid endometrial adenocarcinoma	**2**	**IA**	**6**	**Absent**
A135	Mixed cell carcinoma, high-grade (90% high-grade serous carcinoma, 10% endometrioid adenocarcinoma)	**3**	**IA**	**38**	**Absent**
A138	Endometrioid carcinoma	**1**	**IB**	**64**	**Absent**
A140	Endometrioid carcinoma	**1**	**IA**	**35**	**Absent**
A142	Endometrioid adenocarcinoma	**2**	**IA**	**10**	**Absent**
A145	Endometrioid adenocarcinoma	**2**	**I**	**25**	**Absent**
A147	Carcinosarcoma (predominantly endometrioid adenocarcinoma FIGO grade III)	**3**	**IA**	**48**	**Present**

Supplementary Table S2: MMR status of PD-L1+ tumor cells (by IHC) versus PD-L1+ TCAFs in patients with endometrial cancers

FFPE	PD-L1+ Patient ID	Primary Culture	MMR Status	Detailed MMR Status			
Tumor-IHC		TCAF-ICC		MLH1	MSH2	MSH6	PMS2
PD-L1+	**EA99-1**	<1%	MMR-proficient	Normal	Normal	Normal	Normal
PD-L1+	**EA101-2**	PD-L1+	dMMR	Normal	Normal	Normal	Abnormal
PD-L1+	**EA102-3**	<1%	MMR-proficient	Normal	Normal	Normal	Normal
PD-L1+	**EA103-4**	<1%	MMR-proficient	Normal	Normal	Normal	Normal
PD-L1+	**EA104-5**	<1%	MMR-proficient	Normal	Normal	Normal	Normal
PD-L1+	**EA107-6**	PD-L1+	MMR-proficient	Normal	Normal	Normal	Normal
PD-L1+	**EA108-7**	PD-L1+	dMMR	Abnormal	Normal	Normal	Abnormal
Not Done	**EA111-8**	PD-L1+	MMR-proficient	Normal	Normal	Normal	Normal
PD-L1+	**EA115-9**	PD-L1+	dMMR	Abnormal	Normal	Normal	Abnormal
PD-L1+	**EA116-10**	PD-L1+	MMR-proficient	Normal	Normal	Normal	Normal
PD-L1+	**EA118-11**	Not Done	MMR-proficient	Normal	Normal	Normal	Normal
PD-L1+	**EA119-12**	Not Done	MMR-proficient	Normal	Normal	Normal	Normal
PD-L1+	**EA124-13**	PD-L1+	MMR-proficient	Normal	Normal	Normal	Normal
PD-L1+	**EA129-14**	PD-L1+	MMR-proficient	Normal	Normal	Normal	Normal
PD-L1+	**EA134-15**	PD-L1+	dMMR	Abnormal	Normal	Normal	Abnormal
Not Done	**EA138-16**	PD-L1+	dMMR	Normal	Normal	Normal	Abnormal
Not Done	**EA142-17**	PD-L1+	dMMR	Abnormal	Normal	Normal	Abnormal
PD-L1+	**EA145-18**	PD-L1+	dMMR	Normal	Normal	Normal	Abnormal
PD-L1+	**EA147-19**	PD-L1+	dMMR	Abnormal	Abnormal	Normal	Abnormal
